# On Local Activity and Edge of Chaos in a NaMLab Memristor

**DOI:** 10.3389/fnins.2021.651452

**Published:** 2021-04-20

**Authors:** Alon Ascoli, Ahmet S. Demirkol, Ronald Tetzlaff, Stefan Slesazeck, Thomas Mikolajick, Leon O. Chua

**Affiliations:** ^1^Faculty of Electrical and Computer Engineering, Institute of Circuits and Systems, Technische Universität Dresden, Dresden, Germany; ^2^Department of Microelectronics, Brno University of Technology, Brno, Czechia; ^3^Nano-electronic Materials Laboratory gGmbH, Dresden, Germany; ^4^Institute für Halbleiter- und Mikrosystemtechnik, Technische Universität Dresden, Dresden, Germany; ^5^Department of Electrical Engineering and Computer Sciences, University of California, Berkeley, Berkeley, CA, United States

**Keywords:** memristors, non-linear device modeling, circuit- and system-theoretic methods, theory of non-linear dynamics, local activity theory, physical principle of the edge of chaos

## Abstract

Local activity is the capability of a system to amplify infinitesimal fluctuations in energy. Complex phenomena, including the generation of action potentials in neuronal axon membranes, may never emerge in an open system unless some of its constitutive elements operate in a locally active regime. As a result, the recent discovery of solid-state volatile memory devices, which, biased through appropriate DC sources, may enter a local activity domain, and, most importantly, the associated stable yet excitable sub-domain, referred to as edge of chaos, which is where the seed of complexity is actually planted, is of great appeal to the neuromorphic engineering community. This paper applies fundamentals from the theory of local activity to an accurate model of a niobium oxide volatile resistance switching memory to derive the conditions necessary to bias the device in the local activity regime. This allows to partition the entire design parameter space into three domains, where the threshold switch is locally passive (LP), locally active but unstable, and both locally active and stable, respectively. The final part of the article is devoted to point out the extent by which the response of the volatile memristor to quasi-static excitations may differ from its dynamics under DC stress. Reporting experimental measurements, which validate the theoretical predictions, this work clearly demonstrates how invaluable is non-linear system theory for the acquirement of a comprehensive picture of the dynamics of highly non-linear devices, which is an essential prerequisite for a conscious and systematic approach to the design of robust neuromorphic electronics. Given that, as recently proved, the potassium and sodium ion channels in biological axon membranes are locally active memristors, the physical realization of novel artificial neural networks, capable to reproduce the functionalities of the human brain more closely than state-of-the-art purely CMOS hardware architectures, should not leave aside the adoption of resistance switching memories, which, under the appropriate provision of energy, are capable to amplify the small signal, such as the niobium dioxide micro-scale device from NaMLab, chosen as object of theoretical and experimental study in this work.

## 1. Introduction

In recent years, both industry and academia have been devoting efforts toward the exploration of new materials for the fabrication of novel devices, which, combining a number of functionalities within a limited physical volume, may allow the circuit implementation of disruptive computing strategies, allowing to keep the integrated circuit performance (IC) trend predicted by Moore (1965) in the years to come, despite scientists/companies attempting to reduce CMOS transistor dimensions further shall inevitably face a progressive technological/economical failure (Global Foundries Ltd., 2018). In this regard, one of the nanotechnologies with the greatest potential for future electronics (Williams, 2017; Zidan et al., 2018) allows the realization of disruptive circuit elements, known as *memory-resistors*, or *memristors* for short (Chua, 1971, 2014, 2015; Chua and Kang, 1976). While the most economically profitable application field of these two-terminal devices is the non-volatile memory sector (Mikolajick et al., 2009; Ielmini and Waser, 2016), their inherently rich dynamical behavior allows to use them alternatively for sensing or processing data. Their peculiar capability to merge a number of different functionalities locally makes them the key nanotechnology enabler toward the future hardware implementation of novel ground-breaking information processing paradigms, including in-memory-computing (Ielmini and Wong, 2018), bio-inspired mem-computing (Di Ventra and Traversa, 2018; Xia and Yang, 2019; Ascoli et al., 2020b,c, 2021; Tetzlaff et al., 2020), and bio-sensing (Tzouvadaki et al., 2016, 2020) strategies.

Moreover, the use of appropriate materials in their fabrication allows to adopt them as basic building blocks of biomimetic neuromorphic circuits (Burr et al., 2015; Yi et al., 2018; Bohaichuk et al., 2019; Fuller et al., 2019; Serb et al., 2020). In this respect, given that the memory and learning capabilities of biological synapses may be rather accurately captured by non-volatile memristor models (Chua, 2013), and that potassium and sodium ion channels in biological axon membranes essentially are volatile memristors (Ascoli et al., 2020a), as formulated in 1952 from Hodgkin and Huxley in a seminal paper (Hodgkin and Huxley, 1952), for which they were awarded the Nobel Prize in Physiology in 1961, and theoretically proved out in 2012 from Chua in a milestone manuscript (Chua et al., 2012), explaining several paradoxes that arose from their erroneous identification as time-varying resistances, we may conclude that resistance switching memories shall definitely play a fundamental role in the development of bio-realistic hardware implementations of the human brain in the incoming years.

Scientists have already highlighted the capability of certain memristor physical realizations, capable to retain the information stored in their states under zero input, and featuring a finely tunable resistance, to mimic accurately the biological functionalities of synapses (Indiveri et al., 2013). Most importantly, in relation to the research work presented in this paper, other real-world memristors, which fail to store data under no power, but operate excellently as selector devices in non-volatile crossbar memory arrays, allowing to address sneak-path current issues (Zidan et al., 2013), share with the potassium and sodium ion channels the capability to amplify infinitesimal fluctuations in energy, a fundamental property also referred to as *local activity* (LA) (Mainzer and Chua, 2013), making them ideal candidates to build reliable electronic implementations of spiking neurons.

In order to study the complex dynamics, which volatile memristors, blessed with the capability to enter the locally active regime, and, most importantly, its sub-regime, known as *edge of chaos* (EOC) (Mainzer and Chua, 2013), where the seed of complexity is actually planted, may induce in circuits, which accommodate them, recurring to the foundations of the LA theory^1^ (Chua, 2005) is absolutely necessary^2^. In this regard, the present manuscript employs concepts from the theory of complexity (Mainzer and Chua, 2013) as well as non-linear circuit-centered (Chua, 1987) and system (Ascoli et al., 2019; Corinto et al., 2020) theory-centered methods to analyze an experimentally validated simple yet accurate model of a micro-scale volatile memristor^3^ from NaMLab gGmbH (Mähne et al., 2013; Wylezich et al., 2014), allowing us to explain how to stabilize an operating point lying on the negative differential resistance (NDR) region of the device DC current-voltage characteristic, and to draw a comprehensive picture of the possible operating modes of the microstructure. It is instructive to observe that gaining a complete understanding of the non-linear dynamics of particular miniaturized resistance switching memories, which are capable to amplify the small signal superimposed on top of an appropriate bias level, e.g., those realized by Hewlett Packard (Pickett and Williams, 2012) or the ones manufactured at the NaMLab facilities and investigated in this manuscript, is a fundamental pre-requirement toward the future development of a systematic and conscious approach to design bio-inspired spiking neural networks, which, employing memristive electronic implementations of biological neurons (Pickett et al., 2013), are expected to reproduce the extraordinarily rich panorama of computing functionalities of the human brain (Pickett and Williams, 2013) beyond the current capabilities of traditional purely CMOS hardware (Chicca et al., 2014).

*This article holds a strong pedagogical role, providing a legacy of theoretical knowledge to the next generation of scientists interested in the design of neuromorphic electronics. To the best of our knowledge, this is the very first time that the application of circuit and system-theoretic methods, supported by experimental validation, allows to draw a complete classification of all the admissible operating domains of a memristor, namely the LP, the LA, and the EOC regimes, under both voltage and current control. Importantly, this work paves the way toward the development of a novel systematic neuromorphic hardware design approach, in which variability-tolerant measures may be consciously taken to prevent its basic components from exiting safe operating modes*.

In regard to the structure of the article, section 2 explores the DC behavior of the voltage-controlled volatile memristor from NaMLab, identifying the conditions that allow to bias the micro-scale device along the NDR region of its DC characteristic, where it is said to operate in the locally active regime. Section 3 introduces a rigorous discussion on the device LA, namely its capability to amplify infinitesimal fluctuations in energy, on the basis of the analysis of its small-signal equivalent circuit model, identifying also the conditions under which the NbO memristor may enter the “pearl” (Chua, 2005) embedded in the LA domain, namely the EOC, and, providing, finally, a complete classification of all the possible operating regimes of the threshold switch. Importantly, on the basis of concordant experimental measurements and model predictions, section 4 clarifies once and for all when and how does the response of the threshold switch to quasi-static stimuli differs from its DC behavior. A brief discussion, pinpointing the significance of this research work for the future development of a systematic method to design artificial neural networks capable to operate according to the same mem-computing principles lying at the basis of the human brain information processing paradigm, is provided in section 5. Conclusions are finally drafted in section 6. An Appendix with supplementary information on the microstructure under our magnifying glass in this work is provided in section 6.

## 2. Exploration of LA and EOC in an NbO Volatile Memristor

The next section introduces a purely mathematical yet accurate model of a micro-scale niobium oxide (NbO) volatile memristor manufactured at NaMLab gGmbH (Mähne et al., 2013; Wylezich et al., 2014). Under the time invariance assumption^4^, each two-terminal circuit element from the class of *voltage-controlled extended memristors* is defined via the following *differential algebraic equation* (DAE) set^5^ (Chua, 2018):

(1) $$\begin{array}{l} \frac{d\text{x}}{dt}=\text{g}\left(\text{x},v_{m}\right), \end{array}$$

(2) $$\begin{array}{l} i_{m}=G\left(\text{x},v_{m}\right)\cdot v_{m}, \end{array}$$

where *v*_*m*_ (*i*_*m*_) stands for the voltage (current) falling across (flowing through) the one-port^6^, $\text{x}≜{\left[x_{1},x_{2},\ldots ,x_{k}\right]}^{\text{T}}\in {\mathbb{R}}^{k}$ denotes in general a *k*-dimensional memory state^7^, while both *state evolution function* **g**(·, ·) : ℝ^*k*^ × ℝ → ℝ^*k*^ and *memductance function G*(·, ·) : ℝ^*k*^ × ℝ → ℝ depend upon state and input variables. Importantly, the inequality *G*(**x**, 0) ≠ ∞ must hold true, i.e., the memductance function has to be finite for zero voltage in order for the model of an extended memristor to capture its *coincident zero-crossing signature*, which establishes that the output of the device always crosses the time axis at the same instants as its input^8^.

In case the memductance function is independent of the voltage stimulus, the DAE set (1) and (2) reduces to

(3) $$\begin{array}{l} \frac{d\text{x}}{dt}=\text{g}\left(\text{x},v_{m}\right), \end{array}$$

(4) $$\begin{array}{l} i_{m}=G\left(\text{x}\right)\cdot v_{m}, \end{array}$$

and the one-port is said to belong to the class of *voltage-controlled generic memristors*.

**Figure 1:**
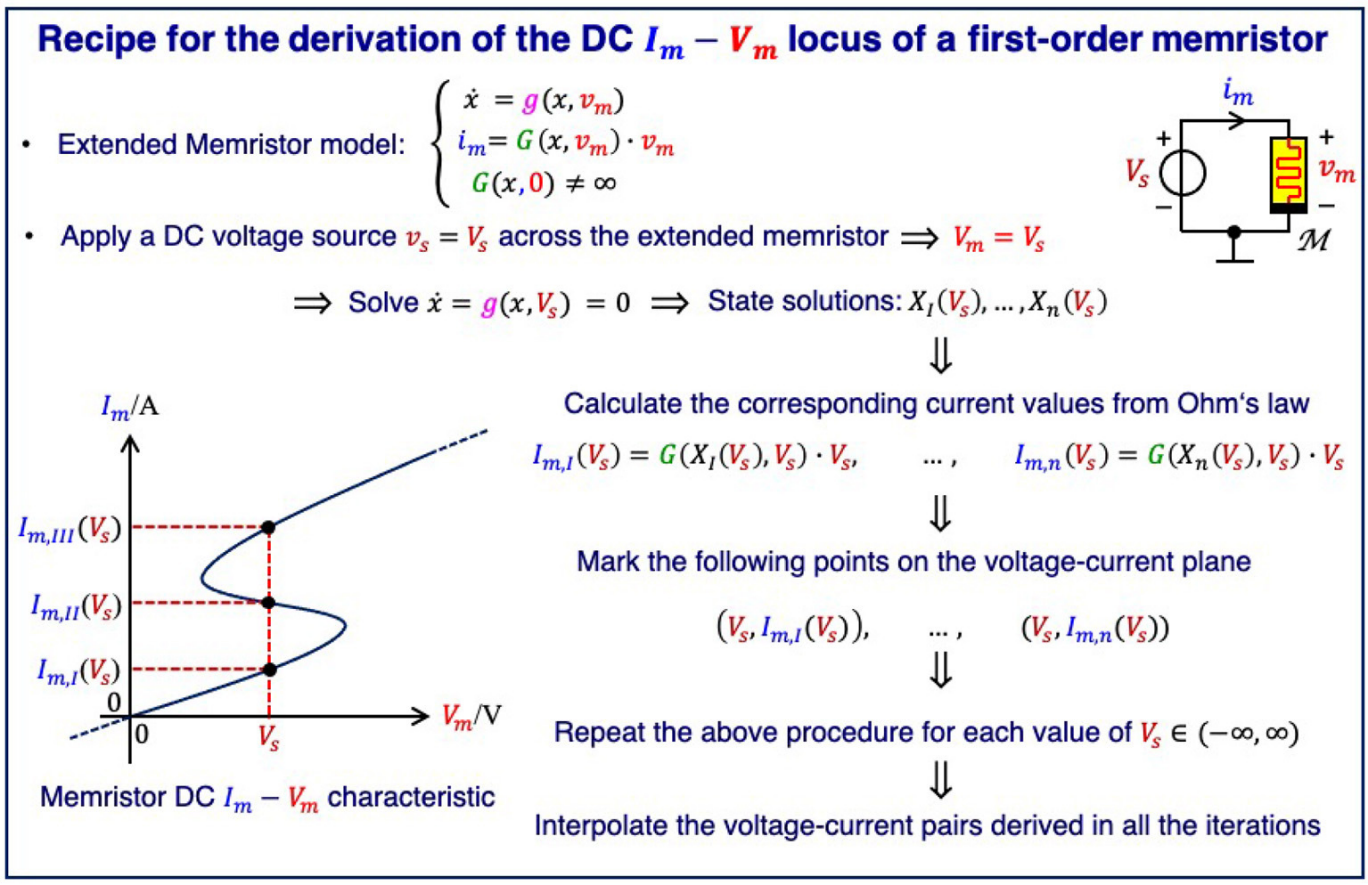
Rigorous method for the determination of the DC current-voltage locus of a first-order voltage-controlled extended memristor.

Figure 1 illustrates the steps necessary to derive the DC current-voltage locus of a first-order voltage-controlled extended memristor^9^ (Chua, 2014) systematically. It is worth to pinpoint that, actually, this rigorous procedure is never carried out in the lab. In fact, among experimentalists, it is common practice to perform *quasi-DC tests* (Chua, 2015), also referred to as *quasi-static measurements*, to characterize the DC current (voltage) response of a voltage (current)-controlled memristor. However, as will be clarified in section 4, extra care should be taken in the selection of the kind of excitation stimulus, i.e., a voltage or a current source, to use for the quasi-static test on a device sample in order to measure a close approximation to its DC characteristic.

### 2.1. Application of a Non-linear System Identification Technique for Modeling a NbO Volatile Memristor

The *Unfolding Theorem*^10^ (Chua, 2011) defines a non-linear system identification method, which allows to determine a purely mathematical description of a memristor device in the voltage-current domain on the basis of a set of experimental data, without requiring a preliminary understanding of the physical mechanisms underlying the device operating principles. Applying this theorem, under low current operation^11^ the non-linear dynamics of a micro-scale NbO volatile resistance switching memory from NaMlab was found to be captured accurately by a voltage-controlled generic memristor model, reading as the DAE set (3) and (4), where the state evolution and memductance functions are respectively, expressed by

(5) $$\begin{array}{l} g\left(x,v_{m}\right)=a_{0}+a_{1}\cdot x+(b_{2}+c_{21}\cdot x+c_{22}\cdot x^{2}+c_{23}\cdot x^{3}\\ \;+c_{24}\cdot x^{4}+c_{25}\cdot x^{5})\cdot v_{m}^{2},\text{ and} \end{array}$$

(6) $$\begin{array}{l} G\left(x\right)=d_{0}+d_{1}\cdot x+d_{2}\cdot x^{2}+d_{3}\cdot x^{3}+d_{4}\cdot x^{4}, \end{array}$$

in which the coefficient values, tuned through a standard optimization procedure, are reported in Table 1.

**Table 1:** Values assigned to the coefficients of the polynomial series developments of state evolution function (5) and memductance function (6) in the proposed NbO micro-device model (3) and (4).

*a*_0_	*a*_1_	*b*_2_	*c*_21_	*c*_22_
5.19 · 10^9^	−2.05 · 10^7^	7.21 · 10^9^	−0.07 · 10^9^	2.27 · 10^5^
*c*_23_	*c*_24_	*c*_25_	*d*_0_	*d*_1_
−2.40 · 10^2^	1.25 · 10^−1^	−2.69 · 10^−5^	6.50 · 10^−3^	−6.66 · 10^−5^
*d*_2_	*d*_3_			*d*_4_
2.14 · 10^−7^	−2.14 · 10^−10^			1.19 · 10^−13^

*A voltage-controlled memristor DAE set (1) and (2), adapted to the first-order case, and with g(x, v_m_) and G(x, v_m_) expressed as sums and products of polynomial series in input and state variables, was chosen as model template. Adopting a bottom-up approach, a standard optimization procedure was set in place to massage the coefficients of the simplest possible form of the polynomial-based model template so as to decrease the cumulative Relative Root Mean Quadratic Error (RRMQE) between a multivariate set of experimental data extracted from a device sample and the corresponding model predictions below a predefined threshold. The complexity of the two series expansions was progressively increased after ensuring that any possible simpler model would fail to meet the objective of the optimization procedure. As compared to the parameter setting, tabulated in Ascoli et al. (2015), where the proposed model was originally formulated, an additional fine tuning of the real coefficients in the polynomial series expansions of g(x, v_m_) and G(x, v_m_) was carried out so as to fit the DAE set to experimental data extracted from a different NbO micro-scale device sample*.

### 2.2. DRM- and Circuit-Theoretic Based Investigations of the Device DC Response

Following the iterative procedure given in Figure 1, the voltage *V*_*s*_ of a hypothetical DC source, inserted in parallel to the NbO device, is varied in small steps from 0 V to 1.046 V, and, for each value of the voltage *V*_*m*_ = *V*_*s*_, which consequently falls across the memristor^12^, the following operations are executed.

- All the possible zeros *X*_*I*_, *X*_*II*_, …, and *X*_*n*_ of the associated state evolution function *g*(*x, V*_*s*_) in Equation (5) are first calculated.
- The *n* memory state DC values are then inserted into Ohm's law (4) to obtain the associated currents *I*_*m,I*_ = *G*(*X*_*I*_) · *V*_*s*_, *I*_*m,II*_ = *G*(*X*_*II*_) · *V*_*s*_, …, and *I*_*m,n*_ = *G*(*X*_*n*_) · *V*_*s*_, where the memductance function is expressed by Equation (6).
- The *n* DC voltage-current pairs (*V*_*s*_, *I*_*m,I*_), (*V*_*s*_, *I*_*m,II*_), …, and (*V*_*s*_, *I*_*m,n*_) are subsequently plotted on the voltage-current plane.

Interpolating all the points, obtained through the entire cycle of iterations, delivers, finally, the DC characteristic of the micro-scale device.

**Figure 2:**
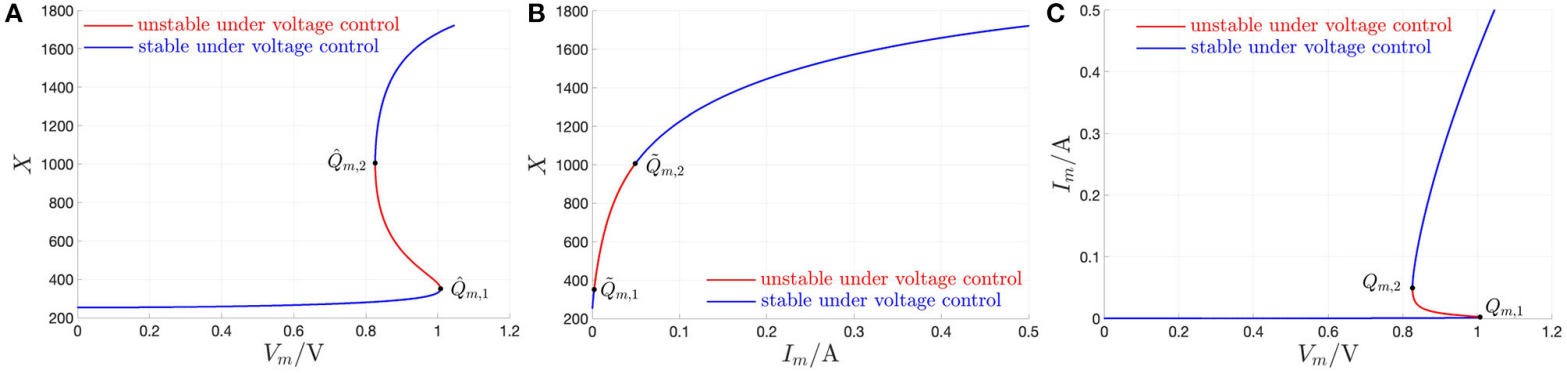
Application of the recipe shown in Figure 1 to obtain the DC *I*_*m*_ vs. *V*_*m*_ locus of the NbO memristor through a voltage sweep. **(A,B)** Any possible zero *X* of the state evolution function *g*(*x, v*_*m*_) for each DC value *V*_*m*_ of the memristor voltage *v*_*m*_ from a set of equally spaced points chosen within the range [*V*_*m,a*_, *V*_*m,b*_] = [0 V, 1.046 V], vs. *V*_*m*_ (*I*_*m*_ = *G*(*X*) · *V*_*m*_). The *X*- and *I*_*m*_-ranges are [*X*_*a*_, *X*_*b*_] = [253, 1722] and [*I*_*m,a*_, *I*_*m,b*_] = [0 A, 500 mA], respectively. **(C)** DC *I*_*m*_ vs. *V*_*m*_ locus obtained by plotting any admissible pair (*V*_*m*_, *I*_*m*_ = *G*(*X*) · *V*_*m*_) inferrable from **(A,B)**. As explained in detail in the text, each point (*V*_*m*_, *I*_*m*_), lying on the NDR region of the DC current-voltage characteristic, obtained under voltage sweep, is found to be unstable. The red and blue colors in **(A–C)** highlight unstable and stable DC operating points of the memristor under voltage control, respectively. The unstable *X*-range is [*X*_1_, *X*_2_] = [351, 1006]. Correspondingly, the memristor voltage and current, respectively, lie within the ranges *V*_*m*_ ∈ [*V*_*m*,2_, *V*_*m*,1_] = [0.826 V, 1.007 V], and *I*_*m*_ ∈ [*I*_*m*,1_, *I*_*m*,2_] = [2.037 mA, 49.296 mA]. The points ${\hat{Q}}_{m,1}=\left(V_{m,1},X_{1}\right)$ and ${\hat{Q}}_{m,2}=\left(V_{m,2},X_{2}\right)$, respectively located at the lower and upper bound of the NDR region in the *V*_*m*_–*X* plane, are shown in **(A)**. The corresponding pair of points in the *I*_*m*_–*X* (*V*_*m*_–*I*_*m*_) plane, namely ${\tilde{Q}}_{m,1}=\left(I_{m,1},X_{1}\right)$ and ${\tilde{Q}}_{m,2}=\left(I_{m,2},X_{2}\right)$ (*Q*_*m*,1_ = (*V*_*m*,1_, *I*_*m*,1_) and *Q*_*m*,2_ = (*V*_*m*,2_, *I*_*m*,2_)), are shown in **(B,C)**.

Figure 2A shows all the possible DC operating points of the memristor state, i.e., all the *X*-values, at which the state equation (3), with state evolution function (5), vanishes, for each *V*_*m*_ value. Correspondingly, using Ohm's law (4), with memductance function (6), the locus of the memory state DC operating point *X* vs. the device DC current *I*_*m*_ is found to be illustrated by Figure 2B. Importantly, *X* is a multi (single)-valued function of the memristor DC voltage (current) for *V*_*m*_ ∈ [*V*_*m,a*_, *V*_*m,b*_] = [0 V, 1.046 V] (*I*_*m*_ ∈ [*I*_*m,a*_, *I*_*m,b*_] = [0 A, 500 mA]), assuming values in the set^13^ [*X*_*a*_, *X*_*b*_] = [253, 1722]. Finally, with reference to Figure 2, on the basis of plots (A) and (B), for each memristor DC voltage value, all the possible DC currents, which may flow through the two-terminal circuit element, are displayed in plot (C), which represents the DC *I*_*m*_–*V*_*m*_ characteristic^14^ of the threshold switch from NaMLab.

Remark 1. *Interestingly, the same results visualized in Figure 2 may be obtained by applying the variant of the recipe in Figure 1, applicable to current-controlled extended memristors, to the current-driven version of the proposed NbO device model DAE set (3) and (4) with g*(*x, v*_*m*_) *and G*(*x*) *expressed by Equations (5) and (6), respectively, i.e., to the current-controlled generic memristor DAE set*

(7) $$\begin{array}{l} \frac{d\text{x}}{dt}=\text{f}\left(\text{x},i_{m}\right), \end{array}$$

(8) $$\begin{array}{l} v_{m}=R\left(\text{x}\right)\cdot i_{m}, \end{array}$$

*where* **f**(·, ·) : ℝ^*n*^ × ℝ → ℝ^*n*^, *denoting the* state evolution function, *and R*(·) : ℝ^*n*^ → ℝ, *standing for the* memristance function, *are respectively, expressed as*

(9) $$\begin{array}{l} f\left(x,i_{m}\right)=g\left(x,G^{-1}\left(x\right)\cdot i_{m}\right),\text{ and } \end{array}$$

(10) $$\begin{array}{l} R\left(x\right)=G^{-1}\left(x\right), \end{array}$$

*and sweeping the memristor DC current I*_*m*_ *within the range*^15^ [*I*_*m,a*_, *I*_*m,b*_] = [0, 500 mA].

**Figure 3:**
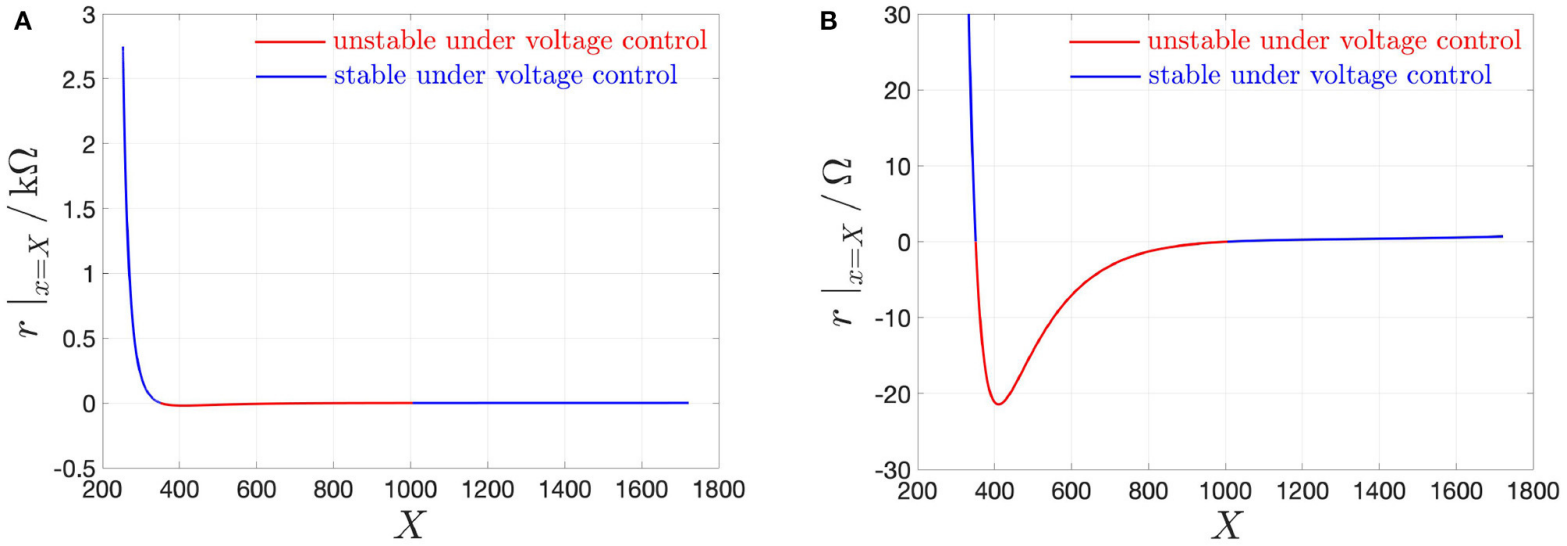
**(A)** Small-signal resistance *r* |_*x*=*X*_ of the NbO memristor vs. memory state DC operating point *X*. In red (blue), the NDR (PDR) values, which, as revealed in the text, are associated to unstable (stable) memory state DC operating points for the voltage-driven memristor device. **(B)** Close-up view on the NDR region, identifiable on the horizontal axis for each *X*-value within the range [351, 1006]. Note that $\hat{r}$, defined in Equation (16), and denoting the largest modulus of the device small-signal resistance in the NDR region, appears at *X* = 411, and is equal to 21.43 Ω. Another state bias point of significance for the remainder of this manuscript is *X* = 478, where *r* is found to be equal to −16.514 Ω.

Calculating the slope of the DC current-voltage locus at each point, which lies along it, the *small-signal or differential or local resistance* $r{|}_{v_{m}=V_{m}}≜{\left({\frac{di_{m}}{dv_{m}}|}_{v_{m}=V_{m}}\right)}^{-1}$ is found to be negative for *V*_*m*_ ∈ (*V*_*m*,2_, *V*_*m*,1_) = (0.826, 1.007) V, and, correspondingly, for *X* ∈ (*X*_1_, *X*_2_) = (351, 1006), and for *I*_*m*_ ∈ (*I*_*m*,1_, *I*_*m*,2_) = (2.037, 49.296)mA. Figure 3A depicts the NbO device small-signal resistance *r* vs. the memory state DC operating point *X*.

The range of *X*-values (*X*_1_, *X*_2_) = (351, 1, 006), where *r* < 0 Ω, defines the *negative differential resistance* (NDR) region of the DC *I*_*m*_–*V*_*m*_ characteristic of the micro-scale threshold switch (Figure 3B). The range of *X*-values [*X*_*a*_, *X*_1_) = [253, 351) ((*X*_2_, *X*_*b*_ = [1, 006, 1, 722]), where *r* > 0 Ω, defines the lower (upper) *positive differential resistance* (PDR) region of the DC *I*_*m*_–*V*_*m*_ characteristic of the micro-scale threshold switch^16^.

Remark 2. *The values of the memristor state in the proposed model reveal an association between *x* and the device internal temperature *T*. On the basis of this assumption, given that the memristor state *X* range along the NDR region, specifically* (*X*_1_, *X*_2_) = (351, 1, 006), *indicates that the threshold switch does not attain the NbO Mott phase transition temperature of T*_*Mott*_ = 1, 050 *K throughout the operating mode where it features a small-signal conductance of negative polarity. This conjecture, examined in Slesazeck et al. (2015), was recently adopted and confirmed in other prominent scientific studies (Gibson et al., 2016; Kumar et al., 2017)*.

A deeper understanding of the device non-linear behavior may be inferred by studying its *dynamic route map* (DRM) (Chua, 2018), a powerful graphical tool for the analysis of first-order dynamical systems^17^.

**Figure 4:**
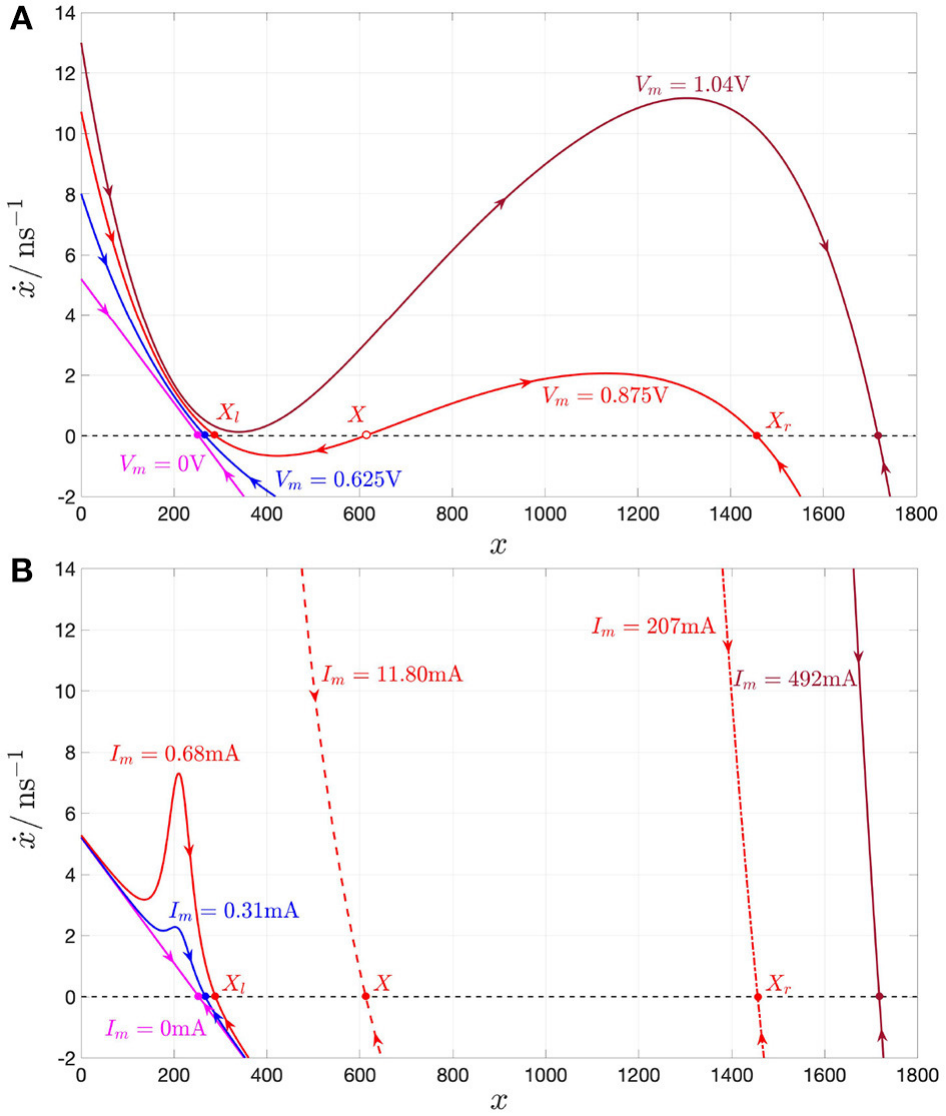
**(A)** DRM of the NbO memristor under voltage control for each *V*_*m*_ value in the set *S*_*V*_*m*__ ≜ {0, 0.625, 0.875, 1.04}V. **(B)** DRM of the NbO memristor under current control for each *I*_*m*_ value in the set *S*_*I*_*m*__ ≜ {0, 0.31, 0.68, 11.80, 207, 492}mA. Analyzing the *V*_*m*_ range [0 V, 1.046 V], shown in the DC *I*_*m*_–*V*_*m*_ characteristic of Figure 2C, the device exhibits a monostable character, except for *V*_*m*_ ∈ [0.826, 1.007 V], where it is found to feature bistability. On the other hand, for each DC current *I*_*m*_ value in the range [0, 500 mA] from Figure 2C, the memristor keeps monostability. In both plots a hollow (filled) circle on the horizontal axis indicates an unstable (a stable) memristor state DC operating point *X* under a given DC input. The pink-colored POP of the device under either voltage or current control crosses the state axis with negative slope at $X=-\frac{a_{0}}{a_{1}}=253.15$, clearly classifying the NbO memristor as a volatile memory according to the proposed model. Also for *V*_*m*_ = 0.625 V, or, alternatively, for *I*_*m*_ = 0.31 mA (for *V*_*m*_ = 1.04 V, or, alternatively, for *I*_*m*_ = 492 mA), the blue (brown) SDR under voltage, or, alternatively, current control, admits a single intersection with the state axis, namely *X* = 267 (*X* = 1725), and negative slope therein. For *V*_*m*_ = 0.875 V, the device features three possible equilibria, namely *X*_*l*_ = 289, *X* = 612, and *X*_*r*_ = 1456, of which only the outer ones are stable, as may be inferred from **(A)**. The memristor may asymptotically attain the first, second, and third operating point in this triplet by letting a specific DC current, equal to 0.68, 11.80, and 207 mA, respectively, flow through the device, as revealed by the red SDR in solid, dashed, and dash-dotted line style in **(B)**.

Figure 4A depicts the NbO device *state dynamic routes* (SDRs) for each value of the memristor DC voltage *V*_*m*_ in the set *S*_*V*_*m*__ ≜ {0, 0.625, 0.875, 1.04} V. As *V*_*m*_ is progressively increased within this set, for the first zero input scenario the voltage-driven memristor exhibits a globally asymptotically stable state operating point. The locus of *ẋ* vs. *x* under no input, known as power-off plot (POP) (Chua, 2015) and depicted in pink in plot (A), crosses in fact the state axis in a single point, namely $X=-\frac{a_{0}}{a_{1}}=253.15$, with negative slope, confirming that, as anticipated earlier, the micro-scale device from NaMLab is a volatile memory under the relatively low current range under focus. The blue SDR, resulting upon the assignment of the second DC value in the set *S*_*V*_*m*__ to *V*_*m*_, also crosses once and with negative slope the state axis, specifically in *X* = 267, and, correspondingly, the memristor keeps its monostable character in this case. As the DC input is further increased, the memristive system loses monostability, acquiring the capability to evolve toward one of two possible operating points. For *V*_*m*_ = 0.875 V, the SDR, drawn in red in plot (A), crosses the memory state axis in three points, specifically *X*_*l*_ = 289, *X* = 612, and *X*_*r*_ = 1, 456, of which each of the outer ones (of which the center one) are locally stable (is unstable), given that $\frac{dẋ}{dx}|{\;}_{V_{m}=0.875\text{ V}}$ is negative (positive) therein. Increasing the DC input further, the device undergoes a reverse bifurcation from bistability back to monostability. For the last *V*_*m*_-value in *S*_*V*_*m*__, the SDR, illustrated in brown in plot (A), admits once again a negative slope in the single location, where it crosses the horizontal axis, namely in *X* = 1, 725, and, as a result, the memristor exhibits one and only one globally asymptotically stable operating point.

Thus, the memristor SDR, resulting upon assigning the first, second, and fourth values (the third value) in the set *S*_*V*_*m*__ to the device DC voltage *V*_*m*_, admits the first, second, and sixth value (the third, fourth, and fifth values) for the memristor state operating point *X* in the set *S*_*X*_ ∈ {253, 267, 289, 612, 1, 456, 1, 725}. From Ohm's law—refer to Equations (4)–(6)—or using directly the data associated with the device DC current-voltage locus in Figure 2C, it may be evinced that the memristor may attain the *k*th equilibrium in the set *S*_*X*_ also upon letting a DC current of value equal to the *k*^*th*^ number in the set *S*_*I*_*m*__ ≜ {0, 0.31, 0.68, 11.80, 207, 492} mA flow across the device (*k* ∈ {1, 2, 3, 4, 5, 6}). Figure 4B depicts the locus of the state evolution function (5), with memristor voltage expressed as $v_{m}=G^{-1}\left(x\right)\cdot i_{m}$, for each DC value *i*_*m*_ = *I*_*m*_ in *S*_*I*_*m*__. With reference to Figure 4, the SDR (all the SDRs), appearing in plot (B), and, respectively, associated with the first, second, and sixth value (respectively, associated with the third, fourth, and fifth values) of the memristor DC current *I*_*m*_ in *S*_*I*_*m*__ is in turn drawn in pink, blue, and brown (are drawn in red), as the SDR, which admits the same equilibrium (admits each of their three distinct equilibria) under voltage control, i.e., the one shown in plot (A), and obtained upon assigning the first, second, and fourth (assigning the third) value in the set *S*_*V*_*m*__ to the memristor DC voltage *V*_*m*_. Remarkably, each of the memristor SDRs, corresponding to values of the device DC current *I*_*m*_ in the set *S*_*I*_*m*__, admits one and only one intersection with the horizontal axis, crossing it with a negative slope, which demonstrates the robust nature of the monostability of the device under current control.

**Figure 5:**
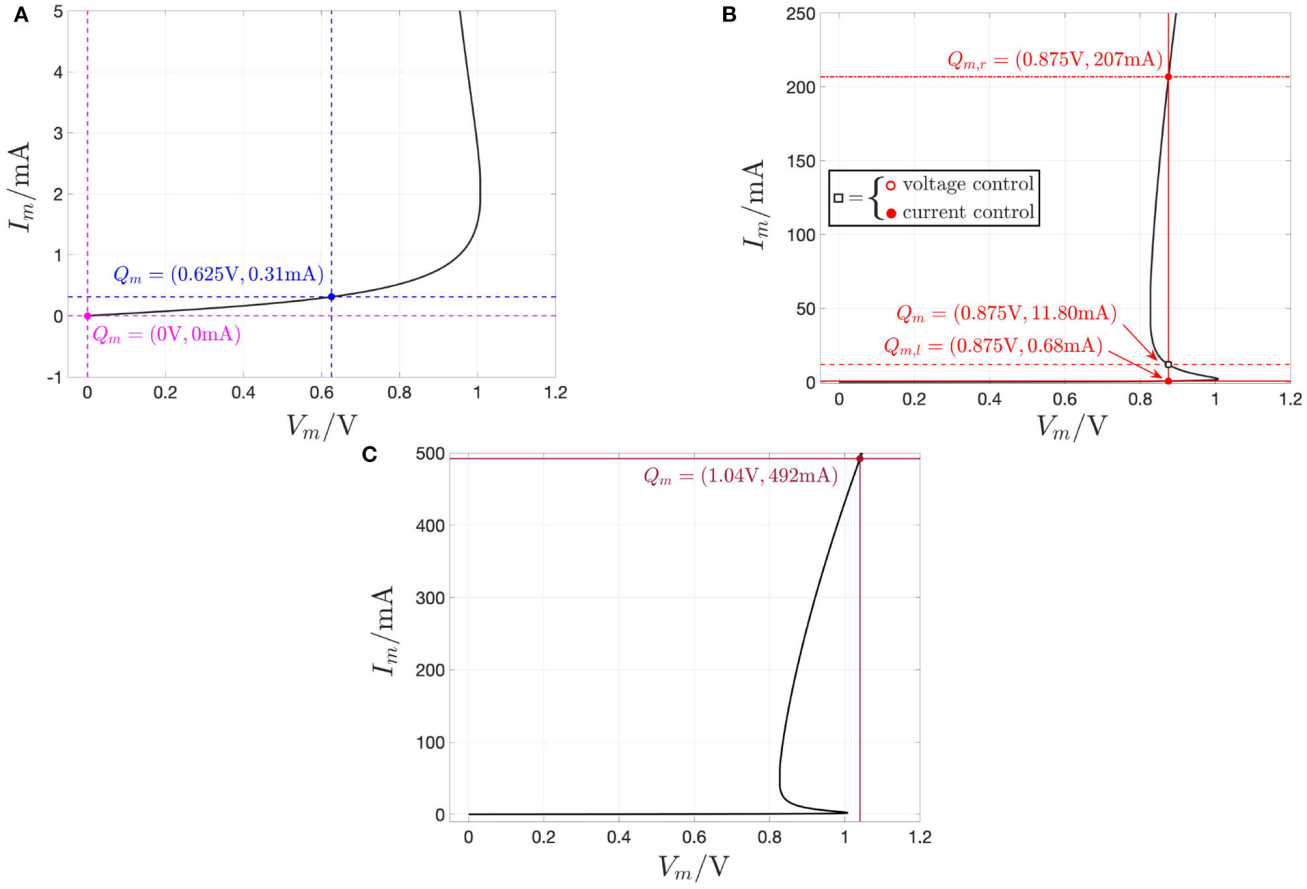
Load line method for the identification of all the possible operating points of the device under any DC voltage (current) input *V*_*m*_ (*I*_*m*_) from the set *S*_*V*_*m*__ = {0, 0.625, 0.875, 1.04}V (*S*_*I*_*m*__ = {0, 0.31, 0.68, 11.80, 207, 492}mA). **(A)** Unique operating point *Q*_*m*_ = (0 V, 0 mA) (*Q*_*m*_ = (0.625 V, 0.31 mA)), marked via a magenta (blue) filled circle, which the black-colored DC *I*_*m*_–*V*_*m*_ locus identifies either with the magenta (blue) vertical load line, resulting from the application of a DC voltage *V*_*s*_ of value 0(0.625)V directly across the memristor, or, alternatively, with the magenta (blue) horizontal load line, associated to the insertion of a current *I*_*s*_ of value 0(0.31)mA into the NbO device. As expected from the magenta (blue) SDR, corresponding to the respective DC voltage or current value, and shown in Figures 4A,B, respectively, the first (latter) operating point is globally asymptotically stable. **(B)** Triplet of operating points, namely *Q*_*m,l*_ = (0.875 V, 0.68 mA), *Q*_*m*_ = (0.875 V, 11.80 mA), and *Q*_*m,r*_ = (0.875 V, 207 mA), denoting the intersections of the black-colored DC *I*_*m*_–*V*_*m*_ locus with the red vertical load line, resulting from the application of a DC voltage of value 0.875 V directly across the memristor. As inferrable from the red SDR in Figure 4A, the lower and upper operating points, marked via red filled circles (the middle operating point, marked via a red hollow circle), are locally stable (is unstable) under voltage control. *Q*_*m,l*_, *Q*_*m*_, and *Q*_*m,r*_ may also be independently set by driving the NbO memristor with a DC current of value 0.68, 11.80, and 207 mA, respectively, as identified by the intersection of the device DC characteristic with the red horizontal load line in solid, dashed, and dash-dotted line style, respectively. As may be evinced by the red solid, dashed, and dashed-dotted SDRs in Figure 4B, the lower, middle, and upper operating point in this triplet is globally asymptotically stable under current control [even *Q*_*m*_ is thus marked via a red filled circle in **(B)**]. **(C)** Unique operating point *Q*_*m*_ = (1.04 V, 492 mA), marked via a brown filled circle, which the black-colored DC *I*_*m*_–*V*_*m*_ locus identifies either with the brown vertical load line, resulting from the application of a DC voltage *V*_*s*_ of value 1.04 V directly across the memristor, or with the brown horizontal load line, associated to the insertion of a current *I*_*s*_ of value 492 mA into the NbO device. As expected from the brown SDR, corresponding to the respective DC voltage or current value, and shown in Figures 4A,B, respectively, this operating point is globally asymptotically stable, irrespective of the nature of the device control signal.

**Figure 6:**
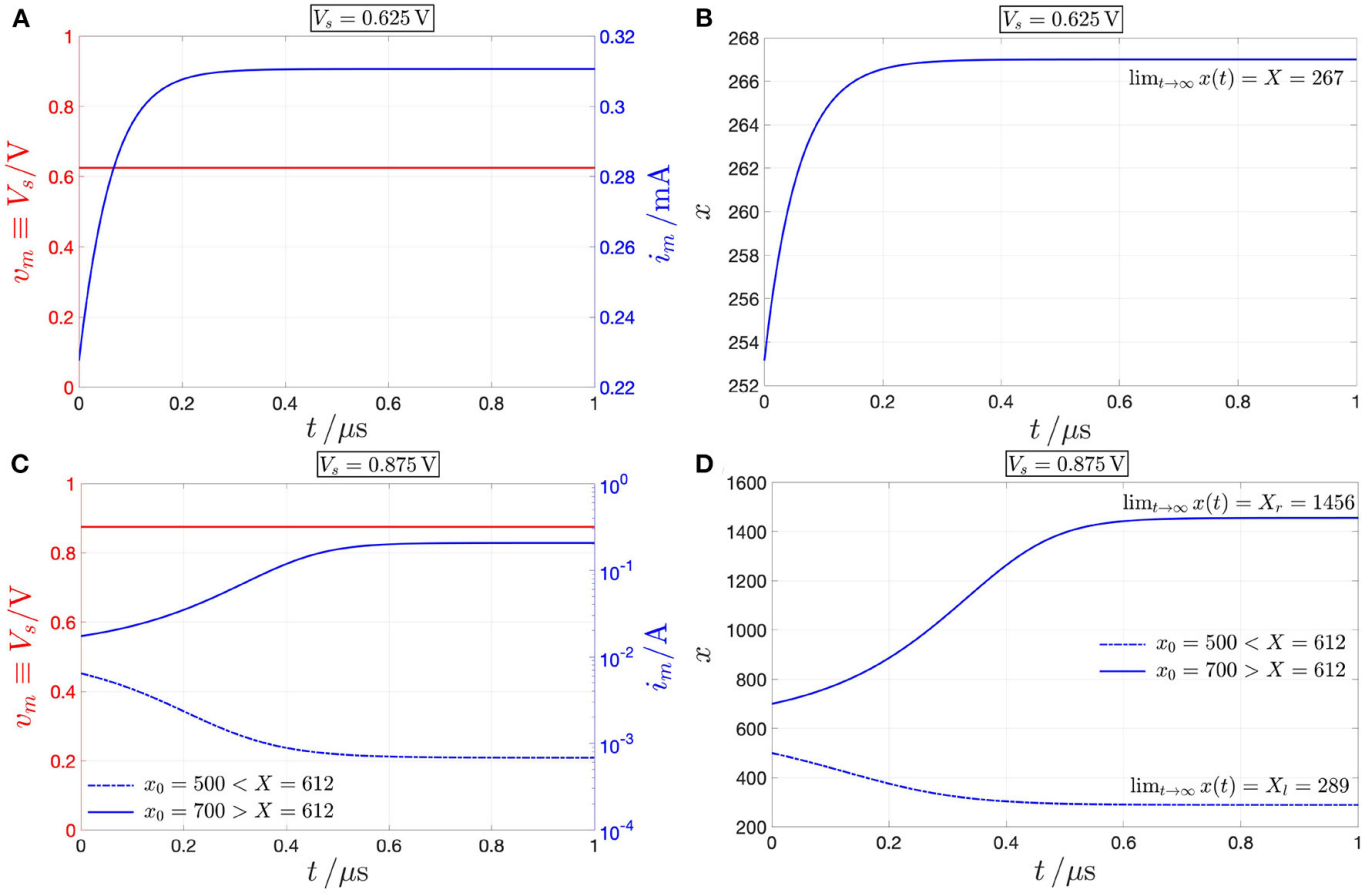
Response of the NbO threshold switch to the application of an independent source of DC voltage *V*_*s*_ between its two terminals. **(A)** Memristor current (blue) under DC voltage stress with *V*_*s*_ = 0.625 V (red). Here, the load line—refer to the vertical blue line in Figure 5A—intersects the memristor DC locus in the lower PDR branch. **(B)** Time waveform of the memristor state, converging to the globally asymptotically stable value *X* = 267 as *t* tends to ∞. **(C)** Dash-dotted (solid) blue waveform: Current through the NbO micro-structure under DC voltage stress with *V*_*s*_ = 0.875 V (red) and for an initial condition *x*_0_ smaller (larger) than the unstable memristor operating state *X* = 612, constituting the separatrix between the basins of attraction of the two-locally stable bias memory states *X*_*l*_ = 289 and *X*_*r*_ = 1456 (recall Figure 4). Here the load line—refer to the vertical red line in Figure 5B—crosses the memristor DC characteristic in the NDR region, where the unstable operating point lies, as well as in either of the two PDR regions, where the threshold switch may be found asymptotically. **(D)** Dash-dotted (solid) blue solution: Time evolution of the memristor state, converging to the locally asymptotically stable value *X*_*l*_ = 289 (*X*_*r*_ = 1, 456) as *t* tends to ∞, for *x*_0_ < (>)*X* = 612.

Remark 3. *A deeper understanding may be gained by analyzing the memristor DC response from a circuit-theoretic perspective. The load line corresponding to the scenario, where a voltage (current) source *V*_*s*_ (*I*_*s*_) is applied directly across the NbO threshold switch, corresponds to a vertical (horizontal) linear locus of equation *V*_*m*_ = *V*_*s*_ (*I*_*m*_ = *I*_*s*_) in the voltage–current plane. Under voltage (current) control, each intersection Q*_*m*_ = (*V*_*m*_ = *V*_*s*_, *I*_*m*_) (*Q*_*m*_ = (*V*_*m*_, *I*_*m*_ = *I*_*s*_)) *between the vertical (horizontal) load line and the memristor DC *I*_*m*_–*V*_*m*_ characteristic is a possible operating point for the device. With reference to Figure 5, for each *V*_*s*_ value in the set* {0, 0.625} *V (for V*_*s*_ = 1.04 *V) the memristor admits a unique operating point *Q*_*m*_, with coordinates reported in plots (A) and (C), and with a globally asymptotically stable character, as clear from the analysis of the respective SDR in Figure 4A. For example, stressing the device with a DC voltage of value V*_*s*_ = 0.625 *V, as shown through a red color in Figure 6A, the threshold switch may be polarized in one and only one globally asymptotically stable operating point, which lies on the lower PDR of its DC *I*_*m*_–*V*_*m*_ characteristic, specifically at Q*_*m*_ = (*V*_*m*_, *I*_*m*_) = (0.625 V, 0.31 mA), *as expected from Figure 5A, and demonstrated through a numerical simulation of the proposed polynomial-based model (see Figures 6A,B, depicting in blue the time evolution of memristor current and state, respectively)*.

*However, as illustrated in Figure 5B, for V*_*s*_ = 0.875 *V, three are the voltage-current pairs, at which the device DC characteristic crosses the respective vertical load line, i.e., *Q*_*m,l*_, *Q*_*m*_, and *Q*_*m,r*_, of which the one (the two) lying on (outside of) the NDR region of the DC *I*_*m*_–*V*_*m*_ locus is unstable (are locally asymptotically stable), as established through the investigation of the respective SDR in Figure 4A. Stimulating the NbO threshold switch by means of a source of DC voltage V*_*s*_ = 0.875 *V, as shown in red in Figure 6C, a couple of numerical simulations of the proposed unfolding theorem-based model provide evidence for the threshold switch bistability. Setting the initial memristor state *x*_0_ below (above) the unstable point X* = 612, *the device may be polarized in a locally asymptotically stable operating point lying on the lower (upper) PDR branch of its DC *I*_*m*_–*V*_*m*_ characteristic, in particular at Q*_*m,l*_ = (0.875 V, 0.68 mA) *[Q*_*m,r*_ = (0.875 V, 207 mA)*], as expected from Figure 5B, and illustrated in Figures 6C,D, depicting in blue and with a dash-dotted (solid) linestyle the approach of memristor current and state to the lower (upper) bias solution from the admissible stable pair, respectively*.

**Figure 7:**
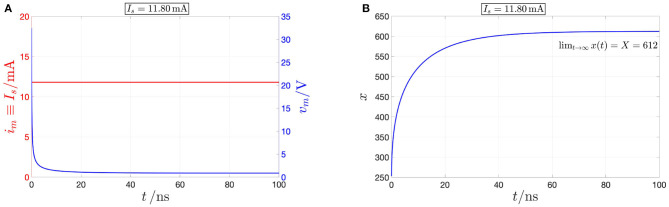
Model numerical simulation demonstrating the inherently stable nature of the NDR operating point *Q*_*m*_ = (*V*_*m*_, *I*_*m*_) = (0.875 V, 11.80 mA) under current control. A DC source of current *I*_*s*_ = 11.80 mA, shown through the red color in **(A)**, is applied directly across the NbO threshold switch. The resulting time waveforms of memristor voltage [in blue in **(A)**] and state [in blue in **(B)**] approach asymptotically the DC values *V*_*m*_ = 0.875 V, and *X* = 612, respectively. The initial condition *x*_0_ was arbitrarily chosen as 253.15.

*On the contrary, referring once more to Figure 5, for each *I*_*s*_ value in *S*_*I*_*m*__—see plots (A), (B), and (C) for I*_*s*_ ∈ {0, 0.31} *mA, for I*_*s*_ ∈ {0.68, 11.80, 207,} *mA, and for I*_*s*_ = 462 *mA, respectively—the device is found to possess one and only one operating point *Q*_*m*_, which is globally asymptotically stable, as determined earlier on through the study of the respective current-driven memristor SDRs shown in Figure 4B. Applying a source of DC current I*_*s*_ = 11.80 *mA directly across the threshold switch, as shown in red in Figure 7A, a numerical simulation of the polynomial-based model demonstrates the global asymptotic stability of the operating point Q*_*m*_ = (*V*_*m*_, *I*_*m*_) = (0.875 V, 11.80 mA), *lying on the NDR branch of the device DC characteristic. Setting the initial condition *x*_0_ arbitrarily to* 253.15, *the device is indeed found to converge asymptotically toward this NDR bias point, as expected from Figure 5B, and visualized in Figures 7A,B, where the memristor voltage (state) is found to approach the value V*_*m*_ = 0.875 *V (X* = 612*) as time goes to* ∞.

### 2.3. NDR Stabilization Under Voltage Control

**Figure 8:**
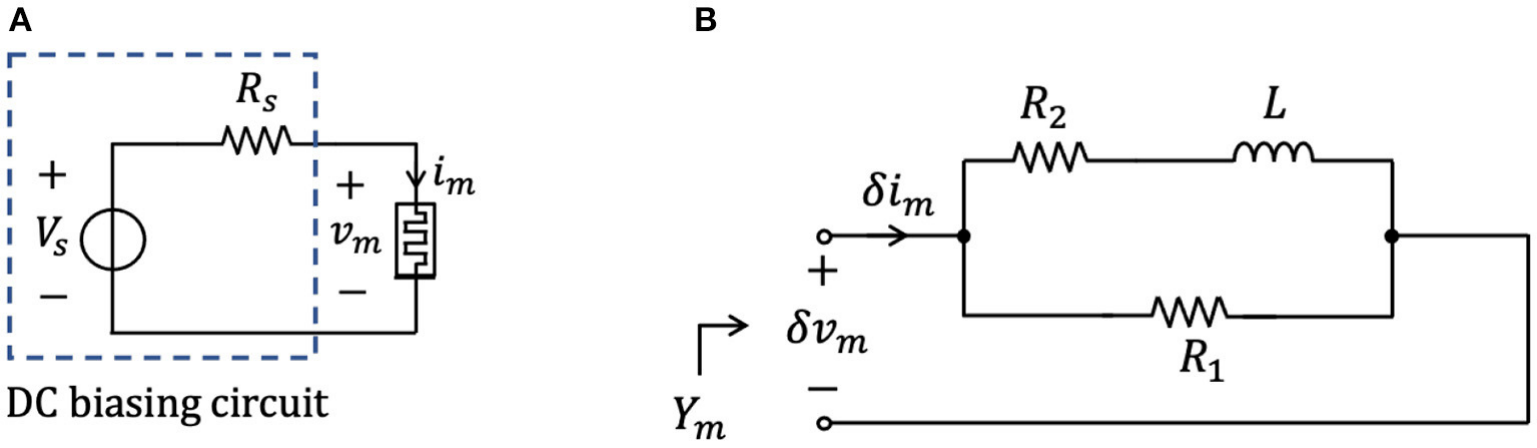
**(A)** Biasing circuit for the stabilization of a given NDR operating point *Q*_*m*_ = (*V*_*m*_, *I*_*m*_) on the threshold switch DC current-voltage characteristic. The introduction of a linear resistor of suitable resistance *R*_*s*_ between the DC voltage source *V*_*s*_ and the threshold switch is instrumental in stabilizing the NDR operating point. As will be clarified shortly, the condition for stabilizing *Q*_*m*_ is *R*_*s*_ > −*r* |_*Q*_*m*__, where *r* |_*Q*_*m*__ denotes the device small-signal resistance at the operating point under focus. **(B)** Small-signal equivalent circuit model of the voltage-controlled NbO memristor. The small-signal input admittance *Y*_*m*_(*s*) of the first-order memristive cell is computed from the port across the device.

**Figure 9:**
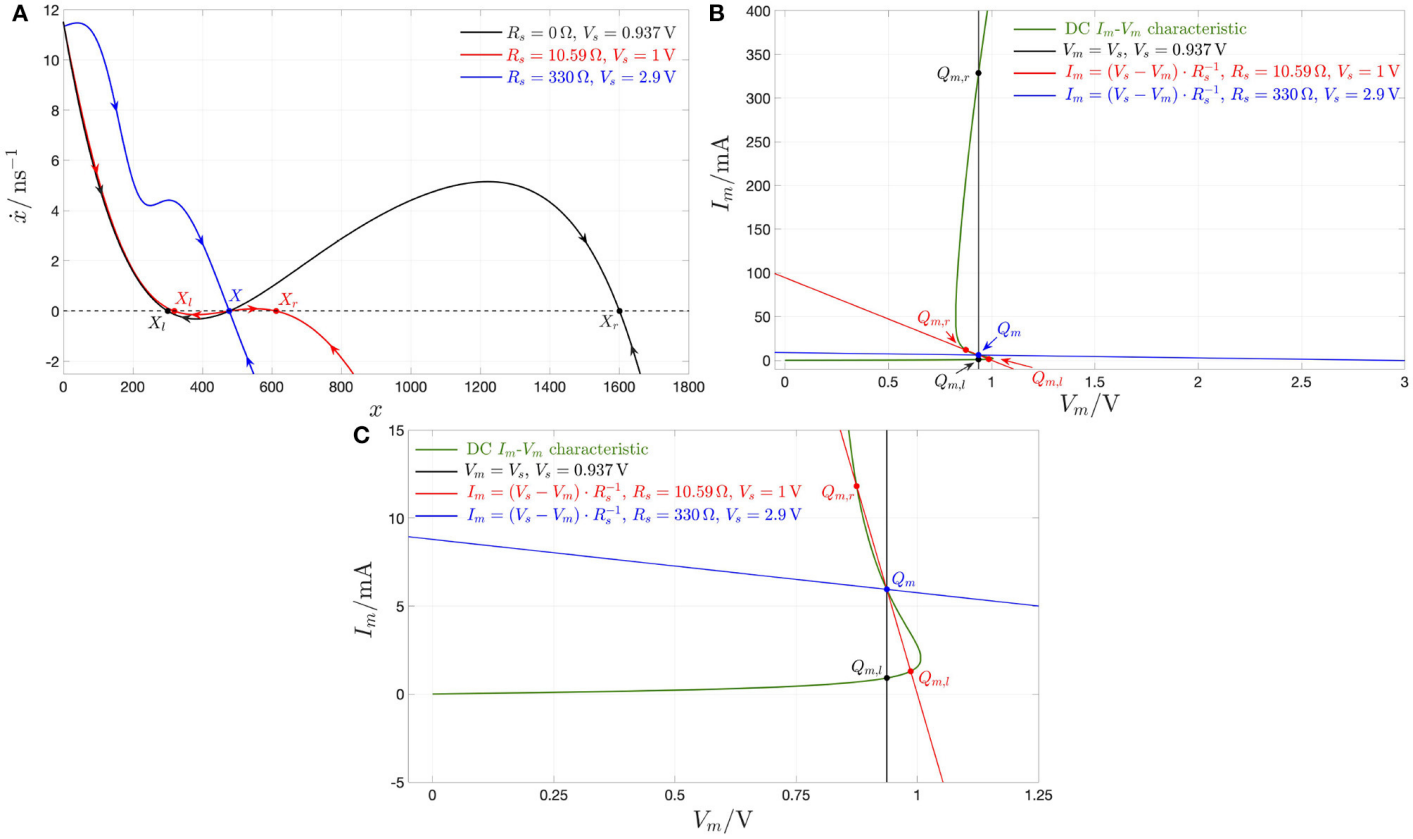
**(A)** Black curve: State Dynamic Route (SDR) of the memristor under the application of a DC voltage *V*_*s*_ = 0.937 V directly across it. Under these circumstances, the NbO device is bistable, with the basins of attraction of the left locally stable state operating point *X*_*l*_, lying at 301, and of the right locally stable state operating point *X*_*r*_, lying at 1, 601, separated by an intermediate unstable state operating point, *X*, lying at 478. Importantly, the memristor voltage *V*_*m*_ and current *I*_*m*_, corresponding to the state operating point *X* = 478, and identifying the coordinates of a NDR point *Q*_*m*_ on the device current-voltage locus, are 0.937 V and 5.948 mA, respectively. Red curve: Memristor SDR under the application of a DC voltage *V*_*s*_ = 1 V in the circuit of Figure 8A with *R*_*s*_ = 10.59 Ω. Under these circumstances, the biasing circuit is unable to stabilize the state operating point *X* = 478, which, similarly as in the previous case, separates the basins of attractions of two locally stable state operating points, i.e., *X*_*l*_ = 319 and *X*_*r*_ = 613, located on its left and right, respectively. Blue curve: SDR of the memristor under the application of a DC voltage *V*_*s*_ = 2.9 V across the series between a linear resistor of resistance *R*_*s*_ = 330 Ω and the memristor itself. In this scenario, the memristor features a single globally asymptotically stable state operating point, lying at the desired location *X* = 478. **(B)** DC current voltage-characteristic of the NbO device (green curve), load line corresponding to the scenario where a DC voltage source of value *V*_*s*_ = 0.937 V is applied directly across the threshold switch (black vertical segment), load line in the case where a DC voltage source of value *V*_*s*_ = 1 V is applied across the series combination between the memristor and a linear resistor with resistance *R*_*s*_ = 10.59 Ω (red negative-sloped segment), and load line for the circuit shown in Figure 8A with *V*_*s*_ = 2.9 V and *R*_*s*_ = 330 Ω (blue negative-sloped segment). Only the stable operating points, appearing in each of the three scenarios, are highlighted, using filled circles, according to the convention adopted in this article. Importantly, the operating point *Q*_*m*_ = (*V*_*m*_, *I*_*m*_) = (0.937 V, 5.948 mA), found to be unstable for (*V*_*s*_, *R*_*s*_) = (0.937 V, 0 Ω) as well as for (*V*_*s*_, *R*_*s*_) = (1 V, 10.59 Ω), becomes globally asymptotically stable for (*V*_*s*_, *R*_*s*_) = (2.9 V, 330 Ω). **(C)** Close-up view of plot (b) in the region around the desired NDR operating point *Q*_*m*_ = (0.937V, 5.948mA).

The analysis of section 2.2 has revealed that the memristor may be biased in any point of the NDR region of its DC *I*_*m*_-*V*_*m*_ locus by letting a suitable DC current flow through it. On the other hand, the entire NDR region of the memristor DC current-voltage characteristic is unstable under voltage control. This notwithstanding, it is possible to stabilize any point along the NDR domain by inserting a linear resistor *R*_*s*_ of appropriate resistance between the DC voltage source *V*_*s*_ and the memristor, as shown in Figure 8A. This section explains how to tune the parameters of the biasing circuit in this figure for the stabilization of a given NDR operating point *Q*_*m*_ = (*V*_*m*_, *I*_*m*_) on the memristor DC *I*_*m*_–*V*_*m*_ characteristic^18^. An example shall be used to illustrate this concept. Suppose it is desirable to stabilize the memristor state equilibrium *X* = 478, i.e., equivalently, the corresponding operating point *Q*_*m*_ = (0.937 V, 5.948 mA) on the device DC characteristic. Let us select the parameters *V*_*s*_ and *R*_*s*_ of the biasing circuit in three distinct ways, and analyze the resulting scenarios. First, applying a DC voltage of value *V*_*s*_ = 0.937 V directly across the threshold switch, i.e., without introducing a series resistor in Figure 8A, the respective voltage-controlled memristor SDR, depicted in black in Figure 9A, and obtained by plotting the state evolution function of Equation (5) with *v*_*m*_ = *V*_*s*_ against the state *x*, admits a triplet of intersections with the horizontal axis, namely *X*_*l*_ = 301, *X* = 478, and *X*_*r*_ = 1, 601, of which the intermediate one is unstable. Let us now insert a series resistance of value *R*_*s*_ = 10.59 Ω between a DC voltage source of value *V*_*s*_ = 1 V and the memristor. Also in this second scenario the memristor exhibits three possible equilibria, specifically *X*_*l*_ = 319, *X* = 478, and *X*_*r*_ = 613, of which the center one is still unstable, as revealed by the positive slope featured therein by the resulting voltage-controlled memristor SDR, drawn in red in Figure 9A, and derived by expressing the memristor voltage *v*_*m*_—refer to the circuit in Figure 8A—as

(11) $$\begin{array}{l} v_{m}\left(x\right)=\frac{1}{1+R_{s}\cdot G\left(x\right)}\cdot V_{s}. \end{array}$$

Finally, let us now increase the resistance *R*_*s*_ of the series resistance up to 330 Ω, and set the value *V*_*s*_ of the DC voltage source to 2.9 V. The SDR, which the voltage-driven threshold switch features in this scenario, is illustrated in blue in Figure 9A. Remarkably, the locus of the time derivative of the state vs. the state itself in this occasion crosses the horizontal axis only once in the location *X* = 478 with a negative slope. Thus, in this third scenario, the parameter setting of the biasing circuit of Figure 8A permits the stabilization of the NDR operating point *Q*_*m*_ under focus. A deeper insight into the stabilization action of the biasing circuit may be gained via its circuit-theoretic analysis. With reference to Figure 9, as shown in plot (B), the load line associated to the first scenario, where (*V*_*s*_, *R*_*s*_) = (0.937 V, 0 Ω), is the black-colored vertical straight line *V*_*m*_ = *V*_*s*_ = 0.937 V, which crosses the memristor DC characteristic in a triplet of points, specifically *Q*_*m,l*_ = (0.937 V, 909 μ*A*), *Q*_*m*_ = (0.937 V, 5.948 mA), and *Q*_*m,r*_ = (0.937 V, 329 mA), of which the intermediate one is associated with the unstable state equilibrium *X* = 478, as results from the analysis of the black-colored voltage-controlled memristor SDR in plot (A). The load line associated to the biasing circuit shown in Figure 8A for *R*_*s*_ ≠ 0 Ω is mathematically described by^19^

(12) $$\begin{array}{l} I_{m}=\frac{V_{s}-V_{m}}{R_{s}}. \end{array}$$

For (*V*_*s*_, *R*_*s*_) = (1 V, 10.59 Ω), referring to the second scenario, the load line, depicted in red in Figure 9B, also crosses the device DC *I*_*m*_–*V*_*m*_ locus in three points, namely *Q*_*m,l*_ = (0.986 V, 1.294 mA), *Q*_*m*_ = (0.937 V, 5.948 mA), and *Q*_*m,r*_ = (0.875 V, 11.82 mA), of which the center one is associated to the unstable state equilibrium *X* = 478, as follows from the investigation of the red-colored voltage-controlled memristor SDR in Figure 9A. With reference to Figure 9, in the third scenario, where (*V*_*s*_, *R*_*s*_) = (2.9 V, 330 Ω), the load line, depicted in blue in plot (B), crosses the device DC *I*_*m*_–*V*_*m*_ locus in a single point, specifically the desired one, i.e., *Q*_*m*_ = (0.937 V, 5.948 mA), which is associated to the globally asymptotically stable state equilibrium *X* = 478, as results from the study of the blue-colored voltage-controlled memristor SDR in plot (A).

Remark 4. *With reference to Figure 9, plot (C) offers a close-up view of plot (B) in the region surrounding the desired bias point Q*_*m*_ = (0.937 V, 5.948 mA) *on the NDR region of the device DC characteristic. It is instructive to pinpoint that, unless what emerges in the first and second scenarios, for* (*V*_*s*_, *R*_*s*_) = (2.9 V, 330 Ω) *the modulus of the negative slope of the load line at *Q*_*m*_ is smaller than the modulus of the negative slope of the memristor DC *I*_*m*_–*V*_*m*_ locus at the same point, i.e.,*

$$\frac{1}{R_{s}}=3.03\text{ mS}<-{\frac{dv_{m}}{di_{m}}|}_{Q_{m}=\left(0.937\text{ V},\;5.948\text{mA}\right)}=60.555\text{ mS}.$$

In fact, the stabilization of a DC operating point *Q*_*m*_ along the NDR region of the memristor DC locus is guaranteed as long as

(13) $$\begin{array}{l} R_{s}>-r{|}_{Q_{m}}, \end{array}$$

*where, as anticipated in section 2.2, r* |_*Q*_*m*__, *the small-signal or differential or local resistance at *Q*_*m*_, is defined as*

(14) $$\begin{array}{l} r{|}_{Q_{m}}≜{\left({\frac{dv_{m}}{di_{m}}|}_{Q_{m}}\right)}^{-1}. \end{array}$$

Choosing the resistance of the series resistor such that

(15) $$\begin{array}{l} R_{s}>\hat{r}, \end{array}$$

with

(16) $$\begin{array}{l} \hat{r}≜{\text{max}}_{\forall Q_{m}\;:\;r{|}_{Q_{m}}<0\Omega }\left\{-r\;{|}_{Q_{m}}\right\} \end{array}$$

denoting the largest modulus of the differential resistance across the NDR region of the device DC characteristic is sufficient to stabilize every operating point along it. With reference to Figure 3, for our NbO threshold switch $\hat{r}=21.43\Omega$ at *X* = 411, and the series resistor resistance adopted most frequently in this article, i.e., *R*_*s*_ = 330 Ω, is large enough to stabilize the entire NDR region of its DC *I*_*m*_–*V*_*m*_ locus.

**Figure 10:**
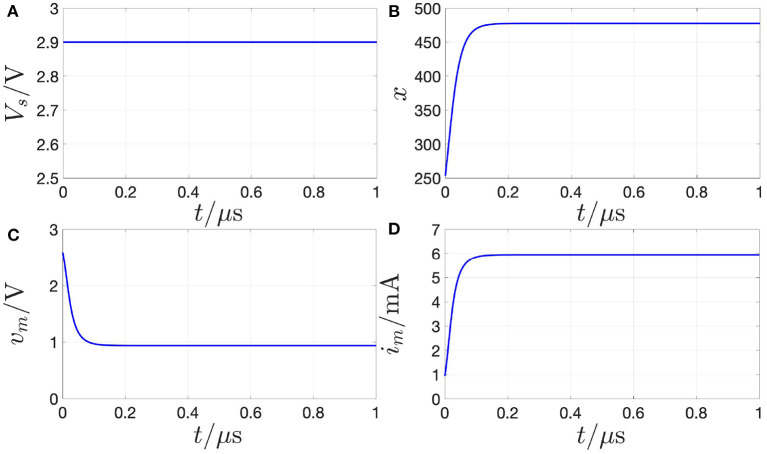
Proof of evidence for the stabilization of the NDR operating point *Q*_*m*_ = (*V*_*m*_, *I*_*m*_) = (0.937 V, 5.948 mA) under voltage control on the basis of a numerical simulation of the polynomial-based model. With reference to the circuit of Figure 8A, a resistor of resistance *R*_*s*_ = 330 Ω is inserted between the source, generating a DC voltage *V*_*s*_ of value 2.9 V [see **(A)**], and the memristor. As shown in **(B–D)**, the memristor state, voltage, and current in turn converge toward the DC values *X* = 478, *V*_*m*_ = 0.937 V, and *I*_*m*_ = 5.948 mA as time goes to infinity. The DAE set initial condition *x*_0_, which may be chosen arbitrarily here, was fixed to 253.15.

Figure 10 demonstrates that, setting the biasing circuit parameters *V*_*s*_ and *R*_*s*_ in the circuit of Figure 8A to 2.9 V, and 330 Ω, respectively, the memristor state, voltage, and current settle asymptotically to the desired values *X* = 478, *V*_*m*_ = 0.937 V, and 5.948 mA, respectively.

## 3. LA and EOC in the NbO Volatile Memristor

Before gaining a deep insight into the conditions under which the NbO threshold switch may enter the LA domain, and, most importantly, its “pearl”-subdomain, i.e., the EOC, let us define rigorously the fundamental concepts of LA and EOC.

### 3.1. LA and EOC: A Rigorous Definition

Without loss of generality, a first-order voltage-controlled^20^ two-terminal memristor device with dynamic state *x* is considered here for introducing this fundamental notion. Let an opportune biasing circuit polarize the memristor in a certain point *Q*_*m*_ = (*V*_*m*_, *I*_*m*_) of its DC current *I*_*m*_-voltage *V*_*m*_ characteristic. Assume that an infinitesimal voltage signal δ*v*_*m*_ is superimposed on the memristor bias voltage *V*_*m*_ at *t* = *t*_0_, generating a total voltage over the device equal to *v*_*m*_ = *V*_*m*_ + δ*v*_*m*_ thereafter. As a result, the device bias state *X* and current *I*_*m*_ also drift by infinitesimal quantities, namely δ*x* and δ*i*_*m*_, respectively, leading to an overall state expressed by *x* = *X* + δ*x*, and to a total current in the form *i*_*m*_ = *I*_*m*_ + δ*i*_*m*_ from the time instant of application of the local perturbation. The memristor is said to be *locally active* at *Q*_*m*_ if and only if it is possible to identify at least one infinitesimal perturbation $\delta v_{m}=\delta v_{m}^{*}$ such that the small-signal or local net energy $\delta E\left(t_{0};t\right)$ absorbed by the device over the time interval [*t*_0_, *t*], and computed via

(17) $$\begin{array}{l} \delta E\left(t_{0};t\right)=\int_{t^{\prime }=t_{0}}^{t^{\prime }=t}\delta v_{m}\left(t^{\prime }\right)\cdot \delta i_{m}\left(t^{\prime }\right)dt^{\prime }, \end{array}$$

by assuming the *associated reference direction convention* for memristor voltage and current^21^ (Chua, 1987), is found to be negative for at least one finite time instant *t* = t^*^. This rigorous definition is however impractical for testing whether a memristor may ever enter the locally active regime, where it may amplify infinitesimal fluctuations in energy. In fact, before concluding that the device is LP across its entire DC current-voltage characteristic, one should ensure that the integral (17) keeps positive at all times and for each of the infinitely many infinitesimal perturbations, which may ever stimulate the memristor, and iterate this procedure for each of the infinitely many one-port bias points. However, and fortunately, there exists a theorem, known as *LA Theorem* (Chua, 2005), which provides necessary and sufficient conditions under which a one-port is locally active at a given operating point *Q*_*m*_. The theorem statement, here adapted to a first-order voltage-controlled^22^ two-terminal memristor device, is enunciated below.

Remark 5. *Under voltage control a first-order memristive one-port is said to be *locally active* at a certain operating point Q*_*m*_ = (*V*_*m*_, *I*_*m*_) *if and only if its local input admittance about *Q*_*m*_ satisfies at least one of 4 conditions. Defining the local transfer function of the voltage-controlled one-port about Q*_*m*_ *as H*_*m*_(*s*) ≜ *Y*_*m*_(*s*), *where* $Y_{m}\left(s\right)=\frac{L\left\{\delta i_{m}\left(t\right)\right\}}{L\left\{\delta v_{m}\left(t\right)\right\}}$ *denotes the* device small-signal admittance, *the 4 conditions*^23^ *may be expressed as follows*^24^:

1. *Y*_*m*_(*s*) *has a single pole s* = *s*_*p*,*Y*_*m*__ *on the right half of the complex plane (RHP), i.e.*, ℜ{*s*_*p*,*Y*_*m*__} > 0.
2. *Y*_*m*_(*s*) *has a single pole s* = *s*_*p*,*Y*_*m*__ = *jω*_*p*,*Y*_*m*__ *lying on the imaginary axis, i.e.,* ℜ{*s*_*p*,*Y*_*m*__} = 0, *and featuring an either complex-valued or negative real-valued residue*^25^.
3. *Y*_*m*_(*s*) *has a pole s* = *s*_*p*,*Y*_*m*__ = *jω*_*p*,*Y*_*m*__ *of order m* > 1 *and located on the imaginary axis*^26^.
4. $Y_{m}\left(j\omega \right)+Y_{m}^{*}\left(j\omega \right)<0$ *for at least one non-negative real-valued angular frequency ω* = *ω*_0,*Y*_*m*__.

*If and only if one and only one of the four conditions in this list, particularly the last one, holds true, the one-port is locally active around an asymptotically stable operating point *Q*_*m*_. In this case, the locally active one-port is said to be on the EOC, which has been dubbed the “pearl” of the LA domain in Chua (2005). If none of the four conditions listed above applies, the one-port is said to be LP at the given operating point*.

### 3.2. Small-Signal Equivalent Circuit Model of the NbO Memristor

This section intends to derive the small-signal equivalent circuit model of the voltage-controlled threshold switch to allow the determination of its local admittance function *Y*_*m*_(*s*). Let us indicate with *X* the state operating point of the memristor under a DC voltage stimulus *V*_*m*_ falling between its terminals, and with *I*_*m*_ the resulting current flowing through the circuit element. With δ*x* ≜ *x* − *X* denoting an infinitesimal change in the memristor state *x* with respect to its operating point *X*, resulting from the application of a small-signal perturbation δ*v*_*m*_ in addition to the DC bias voltage *V*_*m*_ across its terminals, the linearization of state equation (3) and Ohm's law (4), with state evolution and memductance functions expressed by Equations (5) and (6), respectively, provides the following small-signal or local model for the NbO-based memristor:

(18) $$\begin{array}{l} \frac{d\delta x}{dt}=a\cdot \delta x+b\cdot \delta v_{m} \end{array}$$

(19) $$\begin{array}{l} \delta i_{m}=c\cdot \delta x+d\cdot \delta v_{m} \end{array}$$

where δ*i*_*m*_ stands for a local variation in the memristor current with respect to the DC current *I*_*m*_ due to the small-signal voltage input δ*v*_*m*_, while the formulas for the local memristor model coefficients *a*, *b*, *c*, and *d*, respectively, are

(20) $$\begin{array}{l} a={\frac{\partial ẋ\left(x,v_{m}\right)}{\partial x}|}_{\left(x,v_{m}\right)=\left(X,V_{m}\right)}\\ \;=a_{1}+(c_{21}+2\cdot c_{22}\cdot X+3\cdot c_{23}\cdot X^{2}+4\cdot c_{24}\cdot X^{3}\\ \;+5\cdot c_{25}\cdot X^{4})\cdot V_{m}^{2}, \end{array}$$

(21) $$\begin{array}{l} b={\frac{\partial ẋ\left(x,v_{m}\right)}{\partial v_{m}}|}_{\left(x,v_{m}\right)=\left(X,V_{m}\right)}\\ \;=2\cdot (b_{2}+c_{21}\cdot X+c_{22}\cdot X^{2}+c_{23}\cdot X^{3}+c_{24}\cdot X^{4}\\ \;+c_{25}\cdot X^{5})\cdot V_{m}, \end{array}$$

(22) $$\begin{array}{l} c={\frac{\partial i_{m}\left(x,v_{m}\right)}{\partial x}|}_{\left(x,v_{m}\right)=\left(X,V_{m}\right)}\\ \;=(d_{1}+2\cdot d_{2}\cdot X+3\cdot d_{3}\cdot X^{2}\\ \;+4\cdot d_{4}\cdot X^{3})\cdot V_{m},\text{ and } \end{array}$$

(23) $$\begin{array}{l} d={\frac{\partial i_{m}\left(x,v_{m}\right)}{\partial v_{m}}|}_{\left(x,v_{m}\right)=\left(X,V_{m}\right)}\\ \;=G\left(X\right)=d_{0}+d_{1}\cdot X+d_{2}\cdot X^{2}+d_{3}\cdot X^{3}+d_{4}\cdot X^{4}. \end{array}$$

Taking the Laplace transform of each side in the local form of the state equation (18), with *x*(0) = 0, as well as of Ohm's law (19), we obtain:

(24) $$\begin{array}{l} s\cdot L\left\{x\left(t\right)\right\}=a\cdot L\left\{x\left(t\right)\right\}+b\cdot L\left\{v_{m}\left(t\right)\right\} \end{array}$$

(25) $$\begin{array}{l} L\left\{i_{m}\left(t\right)\right\}=c\cdot L\left\{x\left(t\right)\right\}+d\cdot L\left\{v_{m}\left(t\right)\right\}. \end{array}$$

Solving Equation (24) for L{x(t)}, and inserting the resulting expression into Equation (25), the admittance-based transfer function *Y*_*m*_(*s*), defining, within the *s*-domain, the current response of the micro-scale device to a small-signal voltage stimulation around the operating point, is found to be expressed by

(26) $$\begin{array}{l} Y_{m}\left(s\right)=\frac{L\left\{i_{m}\left(t\right)\right\}}{L\left\{v_{m}\left(t\right)\right\}}=d\cdot \frac{s-\frac{a\cdot d-b\cdot c}{d}}{s-a}. \end{array}$$

*Y*_*m*_(*s*) represents the *memristor small-signal or local or infinitesimal admittance*. Its formula in Equation (26) may be implemented in circuit-theoretic form via the circuit, shown in Figure 8B, and consisting of the parallel combination between a linear resistor *R*_1_, and the series connection between yet another linear resistor *R*_2_ and a linear inductor *L*. Of course, the values of the parameters of the electrical elements in this figure, showing essentially *the small-signal equivalent circuit model of the NbO memristor*, depend upon the memristor operating point under focus. From basic circuit-theoretic principles, the Laplace domain representation of the admittance of the one-port in Figure 8B is found to be expressed by

(27) $$\begin{array}{l} Y_{m}\left(s\right)=\frac{1}{R_{1}}\cdot \frac{s+\frac{R_{1}+R_{2}}{L}}{s+\frac{R_{2}}{L}}. \end{array}$$

Imposing the equivalence between the formula (27) for *Y* and the analytical expression (26) for *Y*_*m*_ establishes the following constraints on the dependence of the parameters of the circuit of Figure 8B on the memristor bias point^27^:

(28) $$\begin{array}{l} R_{1}=\frac{1}{d}, \end{array}$$

(29) $$\begin{array}{l} R_{2}=-\frac{a}{b\cdot c},\text{ and } \end{array}$$

(30) $$\begin{array}{l} L=\frac{1}{b\cdot c}. \end{array}$$

**Figure 11:**
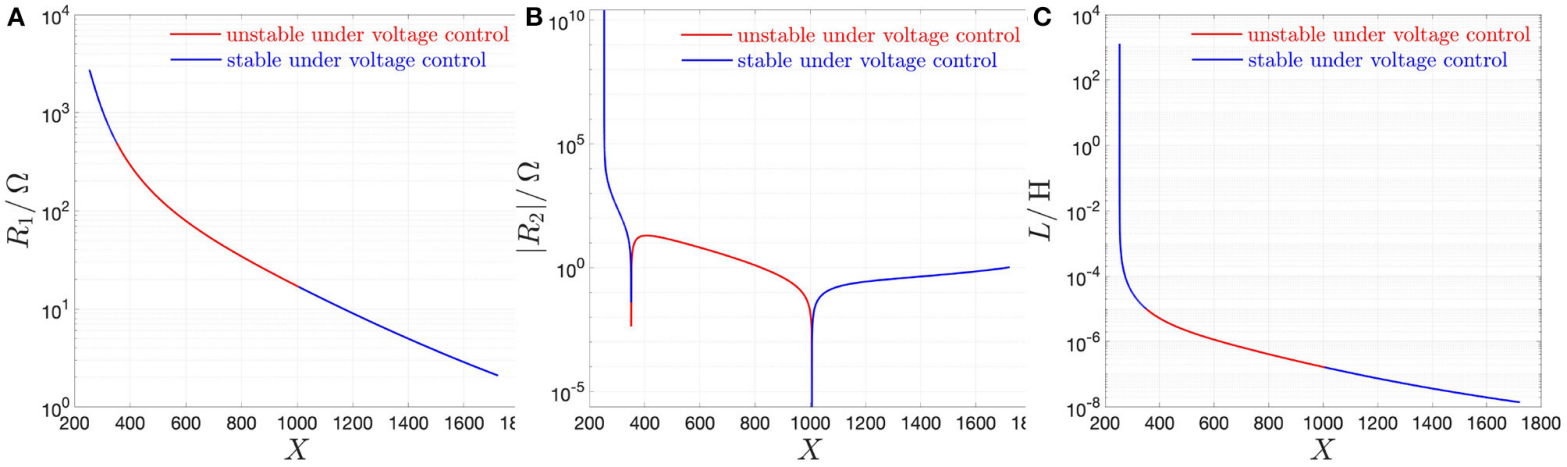
Loci of the resistance *R*_1_ **(A)**, of the modulus of the resistance *R*_2_ **(B)**, and of the inductance *L* **(C)** in the small-signal equivalent circuit model of the NbO threshold switch from NaMLab as a function of the memristor state operating point *X*. In each plot, the red (blue) color is assigned to each branch corresponding to unstable (stable) state operating points under voltage control. As expected, given that it represents the instantaneous resistance *R* = *G*^−1^ of the threshold switch, which is a passive device, *R*_1_ is positive at all operating points. *R*_2_ is found to have a negative sign throughout the red-colored branch, i.e., along the whole NDR region of the memristor DC *I*_*m*_–*V*_*m*_ characteristic (note, however, that *R*_1_ + *R*_2_ is found to be positive at all operating points). Finally, the sign of *L* is positive irrespective of *X*.

Figures 11A–C illustrate the resistance *R*_1_, the modulus of the resistance *R*_2_, and the inductance *L* as a function of the memristor state operating point *X*, respectively. In each plot, the state operating points along the red (blue) branches are unstable (stable) under voltage control, as discussed in section 2.2. Importantly, the resistance *R*_2_ is negative throughout the red branch, i.e., for all *X*-values in the range [351, 1, 006], which corresponds to the entire NDR region of the memristor DC current-voltage characteristic of Figure 2C.

Inserting the expression for *d* from Equation (23) into the formula (28) for *R*_1_, the device instantaneous resistance^28^ *R* ≡ *G*^−1^ may be simply obtained via the resistance of the purely resistive branch in the circuit of Figure 8B, i.e.,

(31) $$\begin{array}{l} R=R_{1}. \end{array}$$

Additionally, the small-signal resistance *r* of the threshold switch may be computed as the parallel combination of the two resistances in the small-signal equivalent circuit model of Figure 8B, i.e.,

(32) $$\begin{array}{l} r=R_{1}||R_{2}. \end{array}$$

*Y*_*m*_(*s*) admits a zero *s*_*z*,*Y*_*m*__ and a pole *s*_*p*,*Y*_*m*__, respectively, located at

(33) $$\begin{array}{l} s_{z,Y_{m}}=-\frac{R_{1}+R_{2}}{L},\text{ and at } \end{array}$$

(34) $$\begin{array}{l} s_{p,Y_{m}}=-\frac{R_{2}}{L}. \end{array}$$

**Figure 12:**
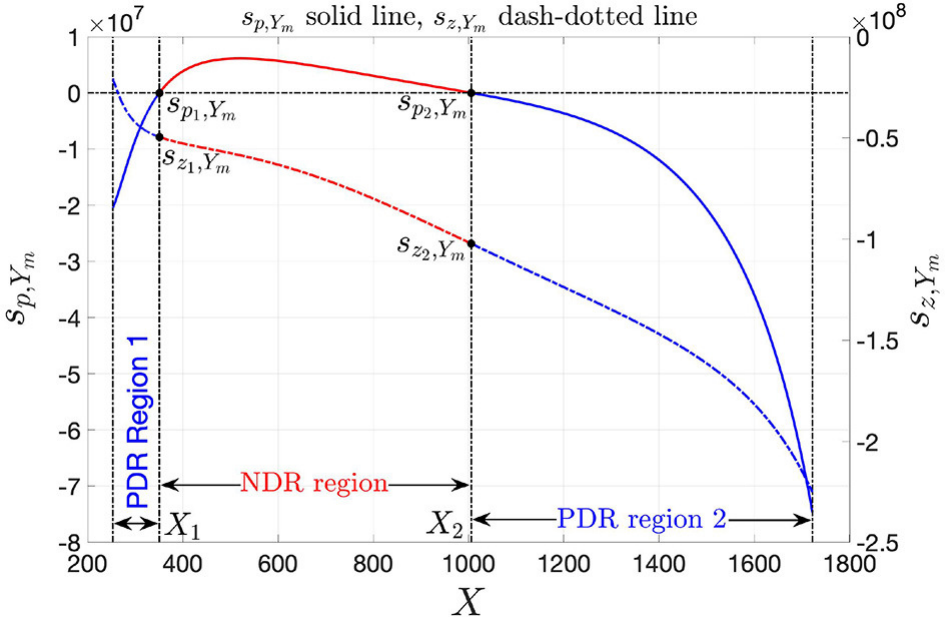
Pole-zero diagram for the local admittance of the voltage-controlled NbO threshold switch from NaMLab GgmbH. While the zero is negative throughout the state operating domain *X* ∈ [*X*_*a*_, *X*_*b*_] = [253, 1, 722] from Figure 2, the pole is negative (positive) in either of the two PDR regions *X* ∈ [*X*_*a*_, *X*_1_) = [253, 351) and *X* ∈ (*X*_2_, *X*_*b*_] = (1, 006, 1, 722] [throughout the NDR region *X* ∈ (*X*_1_, *X*_2_) = (351, 1, 006)] of the threshold switch DC characteristic. Here *s*_*p*_1_,*Y*_*m*__ (*s*_*z*_1_,*Y*_*m*__) and *s*_*p*_2_,*Y*_*m*__ (*s*_*z*_2_,*Y*_*m*__), respectively, are the values of the pole (zero) of *Y*_*m*_(*s*) at the lower *X*_1_ and upper *X*_2_ bounds of the NDR state bias domain.

Figure 12 illustrates the pole-zero diagram of the local admittance *Y*_*m*_(*s*) of the NbO volatile memristor from NaMLab using a blue (red) color in either of the PDR regions (in the NDR region). While the zero from Equation (33), shown through a dash-dotted line, is negative for all the memristor state bias points within the set *X* ∈ [*X*_*a*_, *X*_*b*_] = [253, 1, 722], the pole, located as specified in Equation (34), and depicted by means of a solid line, assumes positive values across the NDR region, i.e., for *X* ∈ (*X*_1_, *X*_2_) = (351, 1, 006), holding the opposite sign in the lower PDR region, i.e., for *X* ∈ [*X*_*a*_, *X*_1_) = [253, 351), and in the upper PDR region, i.e., for *X* ∈ (*X*_2_, *X*_*b*_] = (1, 006, 1, 722].

Remark 6. *The analyses in sections 2.2 and 2.3 have revealed that, without the series resistor, i.e., for R*_*s*_ = 0 Ω *in the circuit of Figure 8A, any bias point Q*_*m*_ = (*V*_*m*_, *I*_*m*_) *lying on the NDR region of the memristor DC current-voltage characteristic is unstable under voltage control. This may be inferred also by observing that the pole *s*_*p*,*Y*_*m*__ of the device local admittance sits on the RHP for each bias point of this kind. Moreover, it is instructive to note that the pole *s*_*p*,*Y*_*m*__ of the device local admittance *Y*_*m*_ coincides with the eigenvalue* λ *of the linearized form of the memristor state Equation (3), with state evolution function expressed by Equation (5), around the same operating point, i.e., recalling that, for R*_*s*_ = 0 Ω *in the circuit of Figure 8A, the voltage *V*_*m*_ across the memristor is just a constant, as established by the DC source*,

(35) $$\begin{array}{l} \lambda ={\frac{dẋ}{dx}|}_{x=X}=a_{1}+c_{21}\cdot V_{m}^{2}+2\cdot c_{22}\cdot V_{m}^{2}\cdot X\\ \;+3\cdot c_{23}\cdot V_{m}^{2}\cdot X^{2}+4\cdot c_{24}\cdot V_{m}^{2}\cdot X^{3}+5\cdot c_{25}\cdot V_{m}^{2}\cdot X^{4}\\ \;\equiv s_{p,Y_{m}}, \end{array}$$

*where the latter equivalence descends from Equations (20), (29), (30), and (34). It follows that the locus of the eigenvalue* λ *vs. the state operating point *X* coincides with the diagram of the pole (34) of the device local admittance *Y*_*m*_(*s*), shown via a solid line in Figure 8*.

*Interestingly, inspecting the formula of the local admittance $Y_{\tilde{m}}$ of the *combined memristor*, consisting of the series combination between the linear resistor *R*_*s*_ and the threshold switch, about the respective operating point* $Q_{\tilde{m}}=\left(V_{\tilde{m}},I_{\tilde{m}}\right)$, *with* $V_{\tilde{m}}=V_{m}+R_{s}\cdot I_{m}$, *and* $I_{\tilde{m}}=I_{m}$, *i.e.*,

(36) $$\begin{array}{l} Y_{\tilde{m}}\left(s\right)=\frac{1}{R_{1}+R_{s}}\cdot \frac{s+\frac{R_{1}+R_{2}}{L}}{s+\frac{R_{s}\cdot \left(R_{1}+R_{2}\right)+R_{1}\cdot R_{2}}{L\cdot \left(R_{1}+R_{s}\right)}}, \end{array}$$

*it is easy to verify that, as expected from the investigation from section 2.2, its pole* $s_{p,Y_{\tilde{m}}}$, *expressed by*

(37) $$\begin{array}{l} s_{p,Y_{\tilde{m}}}=-\frac{R_{s}\cdot \left(R_{1}+R_{2}\right)+R_{1}\cdot R_{2}}{L\cdot \left(R_{1}+R_{s}\right)}, \end{array}$$

*resides on the left half of the complex plane (LHP) as long as the resistance *R*_*s*_ of the series resistor in the biasing circuit shown in Figure 8A is chosen so as to meet the constraint (13), where r*|_*Q*_*m*__ *is the threshold switch small-signal resistance *r*, expressed in (32) as a function of the parameters *R*_1_ and *R*_2_ of the small-signal equivalent circuit shown in Figure 8B, and evaluated at the associated operating point Q*_*m*_ = (*V*_*m*_, *I*_*m*_). *It is worth to point out that the pole $s_{p,Y_{\tilde{m}}}$ of the combined device local admittance $Y_{\tilde{m}}$ about $Q_{\tilde{m}}$ coincides with the eigenvalue $\tilde{\lambda }$ of the linearized form of the memristor state Equation (3), with state evolution function expressed by Equation (5), around the corresponding threshold switch state operating point *X*, i.e., recalling that, for R*_*s*_ ≠ 0 Ω *in the circuit shown in Figure 8A, the voltage across the memristor is a function v*_*m*_ = *v*_*m*_(*x*) *of the memory state *x* as dictated by the voltage divider formula (11)*,

(38) $$\begin{array}{l} \tilde{\lambda }={\frac{dẋ}{dx}|}_{x=X}=\\ \;a_{1}+\left(c_{21}+2\cdot c_{22}\cdot X+3\cdot c_{23}\cdot X^{2}+4\cdot c_{24}\cdot X^{3}+5\cdot c_{25}\cdot X^{4}\right)\cdot v_{m}^{2}\left(X\right)-2\cdot R_{s}\cdot v_{m}^{2}\left(X\right)\\ \;\cdot \frac{\left(b_{2}+c_{21}\cdot X+c_{22}\cdot X^{2}+c_{23}\cdot X^{3}+c_{24}\cdot X^{4}+c_{25}\cdot X^{5}\right)\cdot \left(d_{1}+2\cdot d_{2}\cdot X+3\cdot d_{3}\cdot X^{2}+4\cdot d_{4}\cdot X^{3}\right)}{1+R_{s}\cdot \left(d_{0}+d_{1}\cdot X+d_{2}\cdot X^{2}+d_{3}\cdot X^{3}+d_{4}\cdot X^{4}\right)}\\ \;\equiv s_{p,Y_{\tilde{m}}}, \end{array}$$

*where the last equivalence descends from Equations (20), (21), (22), (23), and (37)*.

### 3.3. Classification of the LP, LA, and EOC Regimes of the NbO Device Under Voltage and Current Control

**Figure 13:**
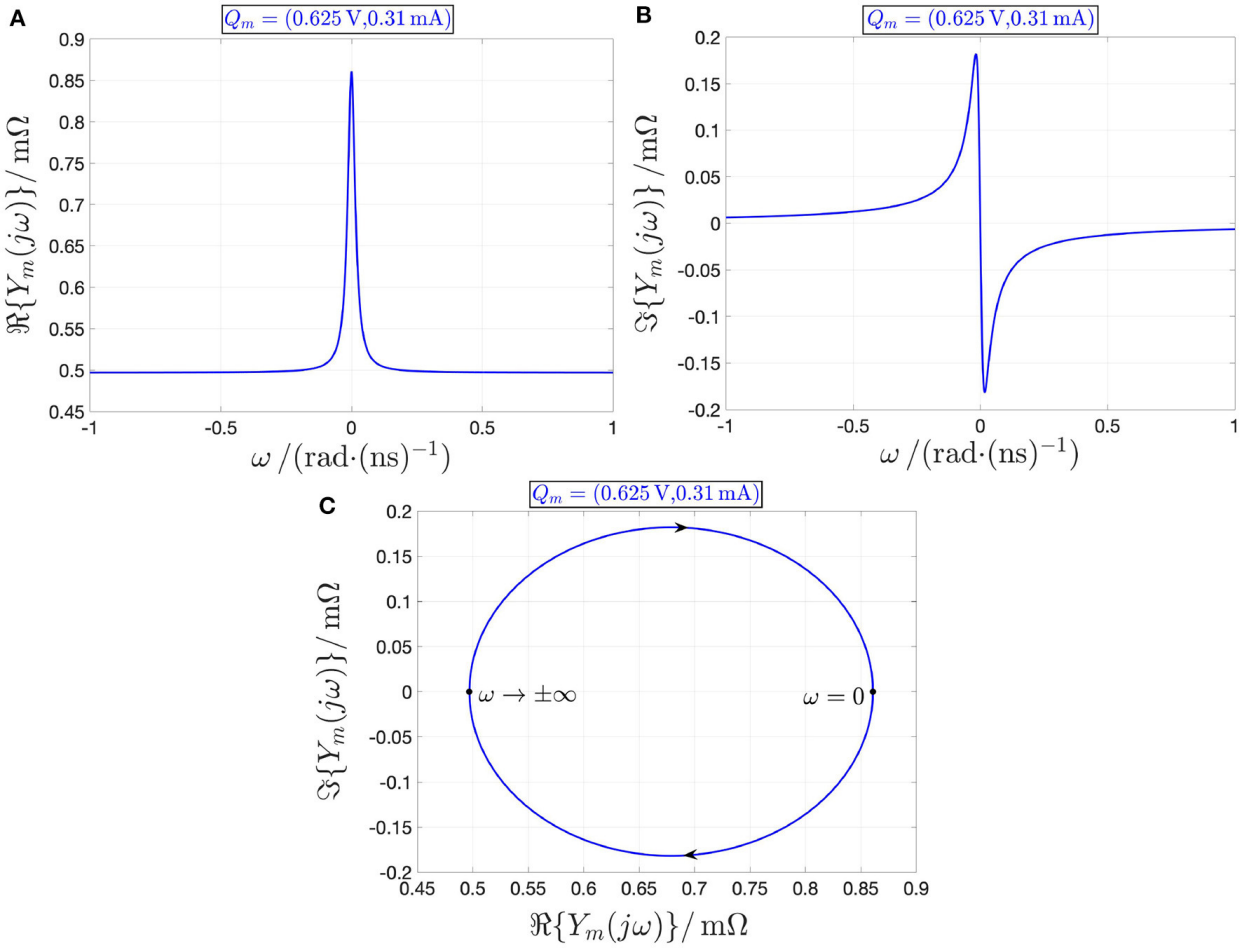
Loci of the real **(A)** and imaginary **(B)** parts of the local impedance *Y*_*m*_(*jω*) of the memristor vs. the angular frequency ω at the operating point *Q*_*m*_ = (*V*_*m*_, *I*_*m*_) = (0.625 V, 0.31 mA). **(C)** Locus of ℑ{*Y*_*m*_(*jω*)} vs. ℜ{*Y*_*m*_(*jω*)}, as computed from **(A,B)**, with arrows showing how the trajectory point evolves along the Nyquist plot as ω increases from −∞ to +∞. The real part of the device local admittance is positive for all angular frequencies. Under voltage control, the threshold switching device is locally passive at the PDR operating point *Q*_*m*_ under focus.

Under voltage control, the pole *s*_*p*,*Y*_*m*__ of the memristor local admittance *Y*_*m*_ about any operating point *Q*_*m*_ = (*V*_*m*_, *I*_*m*_) lying on the NDR region of the one-port DC current-voltage characteristic resides on the RHP, endowing *Q*_*m*_ with an unstable character. The device is locally active at any bias point of this kind, in view of condition (1) from Remark 5. Moreover, in regard to the blue branches in the DC *I*_*m*_–*V*_*m*_ locus of Figure 2C, expressing *Y*_*m*_(*s*) from Equation (27) in the Fourier domain, i.e., as

(39) $$\begin{array}{l} \;Y_{m}\left(j\omega \right)=ℜ\left\{Y_{m}\left(j\omega \right)\right\}+jℑ\left\{Y_{m}\left(j\omega \right)\right\},\text{ with }\\ ℜ\left\{Y_{m}\left(j\omega \right)\right\}=\frac{R_{1}\cdot R_{2}+R_{2}^{2}+\omega^{2}\cdot L^{2}}{R_{1}\cdot \left(R_{2}^{2}+\omega^{2}\cdot L^{2}\right)},\text{ and } \end{array}$$

(40) $$\begin{array}{l} ℑ\left\{Y_{m}\left(j\omega \right)\right\}=-\frac{\omega \cdot L}{R_{2}^{2}+\omega^{2}\cdot L^{2}}, \end{array}$$

given that, as it is easy to demonstrate, ℜ{*Y*_*m*_(*jω*)} is strictly positive therein (see, e.g., Figures 13A–C, respectively, show the loci of ℜ{*Y*_*m*_(*jω*)} vs. ω, ℑ{*Y*_*m*_(*jω*)} vs. ω, and ℑ{*Y*_*m*_(*jω*)} vs. ℜ{*Y*_*m*_(*jω*)} for the state operating point *X* = 267), the voltage-controlled device is stable and locally passive at any PDR bias point, since none of the conditions in Remark 5 holds true.

All in all, as summarized inTable 2, under voltage control the memristor device is locally passive throughout each of the two PDR branches of the *I*_*m*_–*V*_*m*_ locus, while it is locally active and unstable at each operating point on the NDR region of the DC characteristic.

**Table 2:** Complete classification of all the possible operating regimes of the micro-scale device depending upon nature of the stimulus and location of the bias point *Q*_*m*_ = (*V*_*m*_, *I*_*m*_) on the DC characteristic.

**Form of control**	**Location of *Q*_*m*_ on the DC *I*_*m*_–*V*_*m*_ locus**	**Operating regime**
Voltage or current	Either PDR branch	LP
Voltage (current)	NDR branch	Unstable LA (EOC)

*PDR (NDR), positive (negative) differential resistance; LP (LA), local passivity (activity); EOC, edge of chaos. Under current control, the memristor is found to converge toward one and only one globally asymptotically stable operating point Q_m_ for each I_m_ value. Under voltage control, the memristor is found to converge toward one and only one globally asymptotically stable operating point Q_m_ provided the DC stimulus is chosen outside of the NDR range V_m_ ∈ (V_m,2_, V_m,1_) (see the caption of Figure 2 for details). On the other hand, if V_m_ falls within this range, the memristor exhibits bistability, and, depending upon the initial condition, may be asymptotically found in one of two possible locally stable operating points, which lie along distinct PDR branches of the DC current-voltage characteristic.PDR (NDR), positive (negative) differential resistance; LP (LA), local passivity (activity); EOC, edge of chaos. Under current control, the memristor is found to converge toward one and only one globally asymptotically stable operating point Q_m_ for each I_m_ value. Under voltage control, the memristor is found to converge toward one and only one globally asymptotically stable operating point Q_m_ provided the DC stimulus is chosen outside of the NDR range V_m_ ∈ (V_m, 2_, V_m, 1_) (see the caption of Figure 2 for details). On the other hand, if V_m_ falls within this range, the memristor exhibits bistability, and, depending upon the initial condition, may be asymptotically found in one of two possible locally stable operating points, which lie along distinct PDR branches of the DC current-voltage characteristic*.

**Figure 14:**
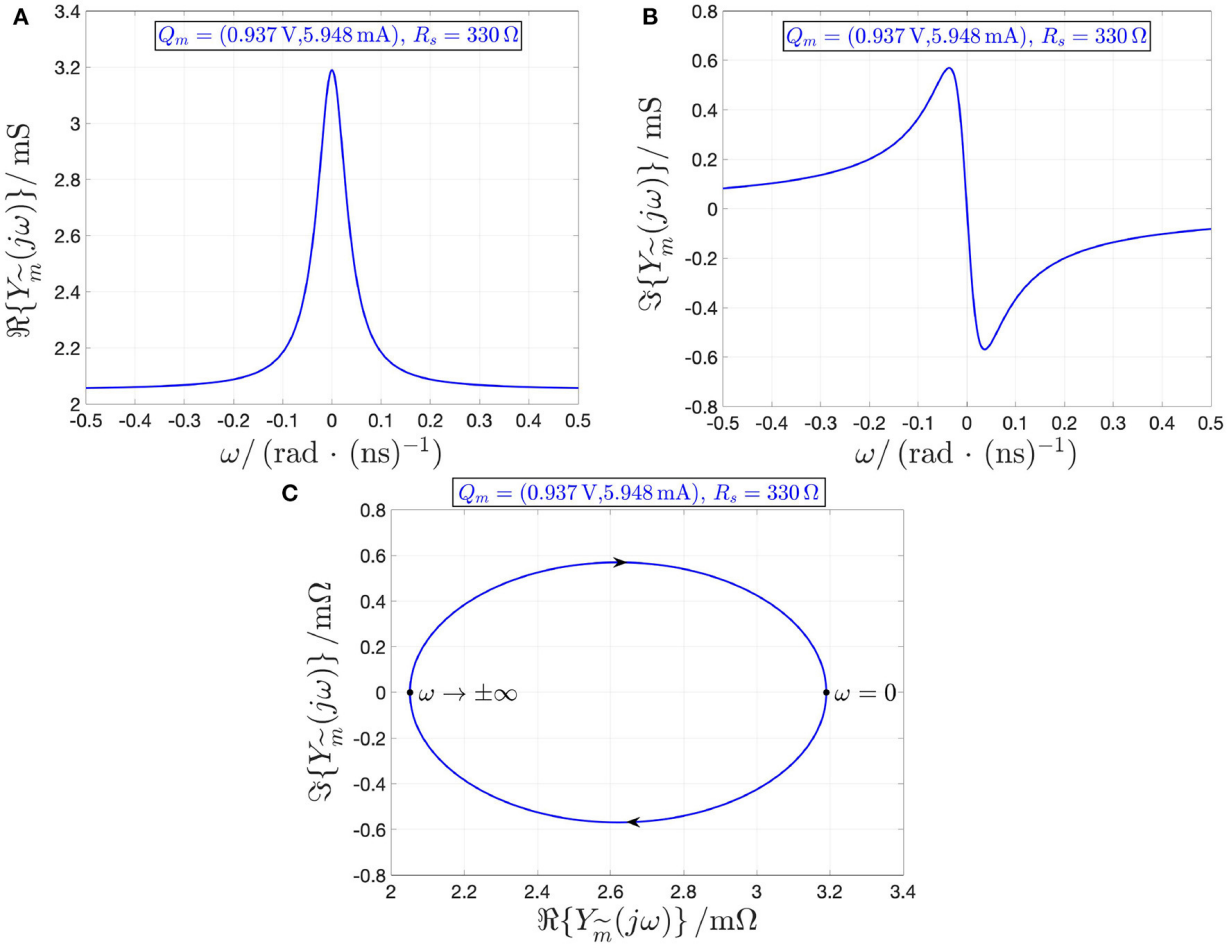
Loci of the real **(A)** and imaginary **(B)** parts of the local admittance $Y_{\tilde{m}}\left(j\omega \right)$ of the combined memristor vs. the angular frequency ω at the operating point $Q_{\tilde{m}}=\left(V_{\tilde{m}},I_{\tilde{m}}\right)=\left(V_{m}+R_{s}\cdot I_{m},I_{m}\right)=\left(2.9\text{ V},5.948\text{ mA}\right)$ for *R*_*s*_ = 330 Ω, *V*_*m*_ = 0.937 V, and *I*_*m*_ = 5.948 mA (at the associated NDR operating point *Q*_*m*_ = (*V*_*m*_, *I*_*m*_), the memristor state operating point *X* amounts to 478). **(C)** Locus of $ℑ\left\{Y_{\tilde{m}}\left(j\omega \right)\right)\}$ vs. $ℜ\left\{Y_{\tilde{m}}\left(j\omega \right)\right\}$, as computed from **(A,B)**, with arrows showing how the trajectory point evolves along the Nyquist plot as ω increases from −∞ to +∞. The real part of the combined device local admittance is positive for all angular frequencies. Under voltage control, the threshold switching device is locally active and unstable at the NDR operating point *Q*_*m*_ under focus, whereas the stabilizing resistance *R*_*s*_, meeting the constraint (13), turns the combined memristor into a locally-passive two-terminal circuit element at the associated operating point $Q_{\tilde{m}}$.

However, as shown in section 3.2, any NDR operating point *Q*_*m*_ = (*V*_*m*_, *I*_*m*_) of the voltage-controlled memristor can be stabilized by inserting a linear resistor, with resistance *R*_*s*_ satisfying the inequality (13), in series with it. In fact, as known in memristor theory (Chua, 1971), this step effectively yields another voltage-controlled memristor, which we called *combined memristor*, and it is composed of the series connection between the resistor and the threshold switch, and is stable at the respective operating point $Q_{\tilde{m}}=\left(V_{\tilde{m}},I_{\tilde{m}}\right)$, where $V_{\tilde{m}}=V_{m}+R_{s}\cdot I_{m}$, and $I_{\tilde{m}}=I_{m}$. Moreover, the voltage-controlled combined memristor is *locally passive* at any bias point $Q_{\tilde{m}}$ associated to a threshold switch operating point *Q*_*m*_, which is stabilized via an opportune choice of the parameters *V*_*s*_ and *R*_*s*_ in the biasing circuit of Figure 8A. This follows from the fact that the real part $ℜ\left\{Y_{\tilde{m}}\left(j\omega \right)\right\}$ of the combined memristor local admittance $Y_{\tilde{m}}\left(j\omega \right)$, which is derived from Equation (36) by replacing the Laplace variable *s* with the Fourier variable *jω*, and is found to be described by

(41) $$\begin{array}{l} Y_{\tilde{m}}\left(j\omega \right)=ℜ\left\{Y_{\tilde{m}}\left(j\omega \right)\right\}+jℑ\left\{Y_{\tilde{m}}\left(j\omega \right)\right\},\text{ with }\\ ℜ\left\{Y_{\tilde{m}}\left(j\omega \right)\right\}\\ =\frac{\omega^{2}\cdot L^{2}\cdot \left(R_{1}+R_{s}\right)+\left(R_{1}+R_{2}\right)\cdot \left(R_{s}\cdot \left(R_{1}+R_{2}\right)+R_{1}\cdot R_{2}\right)}{\omega^{2}\cdot L^{2}\cdot {\left(R_{1}+R_{s}\right)}^{2}+{\left(R_{s}\cdot \left(R_{1}+R_{2}\right)+R_{1}\cdot R_{2}\right)}^{2}},\text{ and} \end{array}$$

(42) $$\begin{array}{l} ℑ\left\{Y_{\tilde{m}}\left(j\omega \right)\right\}=-\frac{\omega \cdot L\cdot R_{1}^{2}}{\omega^{2}\cdot L^{2}\cdot {\left(R_{1}+R_{s}\right)}^{2}+{\left(R_{s}\cdot \left(R_{1}+R_{2}\right)+R_{1}\cdot R_{2}\right)}^{2}}, \end{array}$$

results to be strictly positive across the entire range of real angular frequency values under the constraint (13), which prevents condition (4) from Remark 5 to apply. As an example, Figures 14A,B, respectively, depict the real and imaginary parts of the combined memristor local admittance $Y_{\tilde{m}}\left(j\omega \right)$ about the operating point $Q_{\tilde{m}}=\left(V_{\tilde{m}},I_{\tilde{m}}\right)=\left(2.9\text{ V},5.948\text{ mA}\right)$, corresponding to the threshold switch state operating point *X* = 478 and for the *R*_*s*_ value of 330 Ω, which meets the condition (13). Plotting $Im\left\{Y_{\tilde{m}}\left(j\omega \right)\right\}$ against $Re\left\{Y_{\tilde{m}}\left(j\omega \right)\right\}$, on the basis of these two graphs, yields Figure 14C, where arrows indicate the direction of motion of the trajectory point as ω increases from −∞ to ∞.

**Figure 15:**
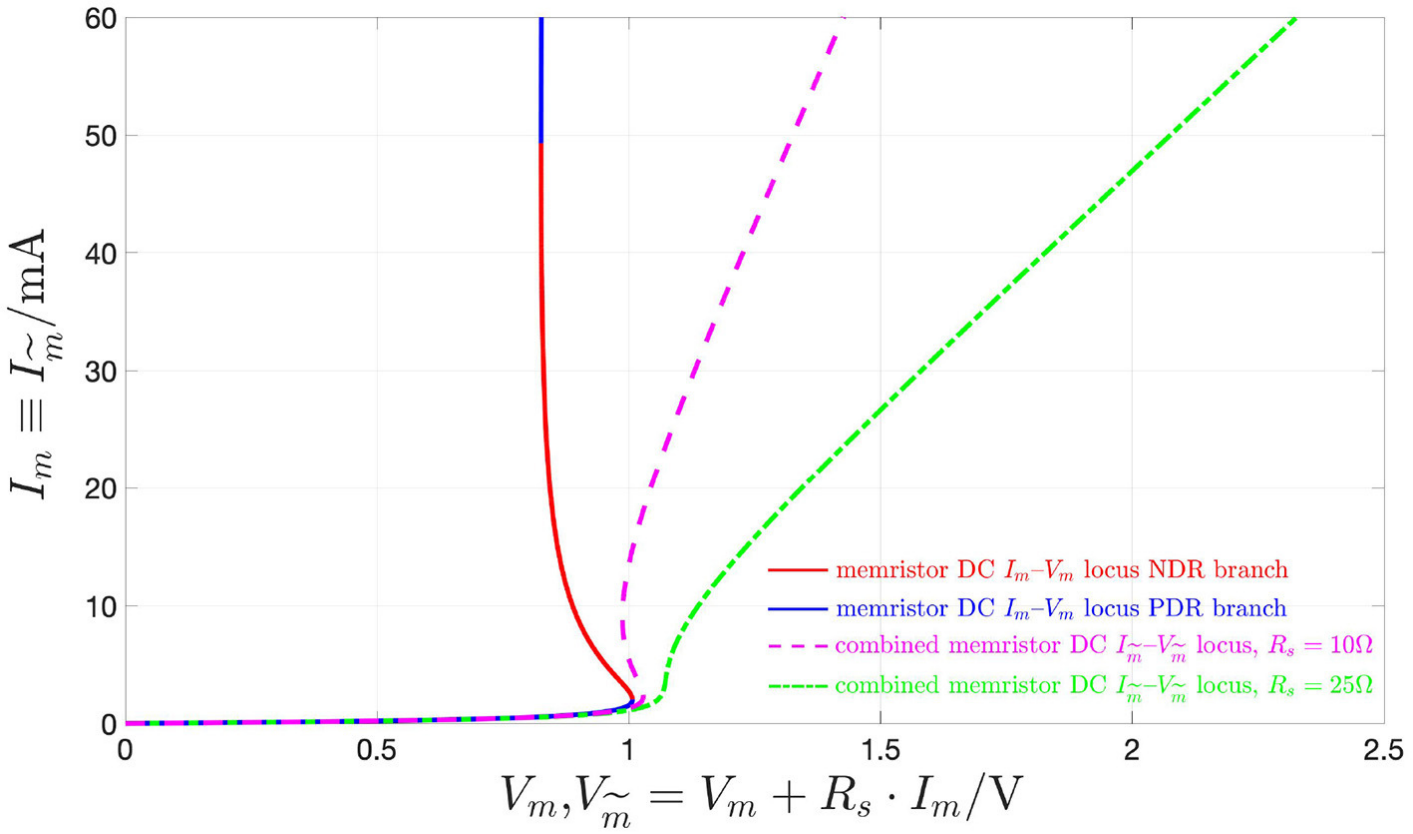
DC current $I_{\tilde{m}}\equiv I_{m}$–voltage $V_{\tilde{m}}=V_{m}+R_{s}\cdot I_{m}$ loci of the combined memristor for *V*_*s*_ = 2.9 V and two distinct valued associated to the resistance *R*_*s*_ of the series resistor, specifically 10 Ω (dashed magenta curve), and 25 Ω (dash-dotted green curve). The DC *I*_*m*_–*V*_*m*_ locus of the threshold switch is also shown for reference using the red (blue) color and the solid line style for the NDR (PDR) branch, which, as studied in section 2.2 is unstable (stable) under voltage control. Note that the small-signal resistance $\tilde{r}$ of the combined memristor, expressed by $\tilde{r}=Y_{\tilde{m}}^{-1}\left(0\right)={\left(R_{s}^{-1}+r^{-1}\right)}^{-1}$, is strictly positive only in the scenario where the value of 25 Ω is assigned to *R*_*s*_. In fact, only in this case *R*_*s*_ satisfies the condition (15), given that, with reference to Figure 3, for our threshold switching device $\hat{r}$, defined in Equation (16), is found to be equal to 21.43 Ω at *X* = 411.

Interestingly, choosing a resistance *R*_*s*_, satisfying the inequality (15), in which for our device $\hat{r}$, as defined in Equation (16), and amounting to 21.43 Ω at *X* = 411, denotes the largest modulus of the threshold switch differential resistance across its entire DC locus NDR region, the combined memristor is found to feature a strictly PDR. This is demonstrated graphically in Figure 15, showing the DC current $I_{\tilde{m}}\equiv I_{m}$-voltage $V_{\tilde{m}}=V_{m}+R_{s}\cdot I_{m}$ characteristics of the combined device in a couple of distinct scenarios, specifically for *R*_*s*_ set to 10 Ω, where the condition (15) is not satisfied (dashed magenta curve), and to 25 Ω, where the constraint (15) is met (dash-dotted green locus), respectively, besides the DC *I*_*m*_–*V*_*m*_ locus of the threshold switch, depicted in solid line style, and using the red and blue colors for the NDR and PDR branches, respectively.

*All in all, under the constraint (15), with* $\hat{r}$ *defined in formula (16), the combined memristor is stable and locally passive along its entire DC* $I_{\tilde{m}}$–$V_{\tilde{m}}$ *locus, since in these circumstances none of the* 4 *conditions from Remark 5 may ever apply at any of its bias points (the green dash-dotted DC current-voltage characteristic, which is illustrated in* *Figure 15* *for R*_*s*_ = 25 Ω*, is exhibited by a combined device of this kind). On the other hand, in case the constraint (13) does not hold true along part of threshold switch NDR branch, then, similarly as it is the case for the threshold switch itself, also the DC characteristic of the voltage-controlled combined memristor would feature a region endowed with negative slope (the magenta dashed DC current-voltage characteristic, which is illustrated in* *Figure 15* *for R*_*s*_ = 10 Ω*, is exhibited by a combined device of this kind), and similar conclusions, as drawn above for the operating regimes of the voltage-controlled NaMLab micro-structure, would apply to the resistor-NbO device series combination under voltage control. More specifically, in these circumstances, the voltage-controlled combined memristor would be locally passive along either of the two PDR branches of the DC* $I_{\tilde{m}}$–$V_{\tilde{m}}$ *locus, while it would be locally active and unstable, in view of condition 1 from Remark 5, throughout its DC characteristic NDR branch, which corresponds to that part of the NDR region of the threshold switch DC I*_*m*_–*V*_*m*_ *locus, where the condition (13) is not met*.

Importantly, as revealed via the investigation of section 2.2, under current control the NbO memristor is stable at each operating point *Q*_*m*_ residing along the NDR region of its DC *I*_*m*_–*V*_*m*_ locus. Here it is of interest to derive the memristor local impedance *Z*_*m*_, which is simply expressed by the inverse of the device local admittance *Y*_*m*_ of Equation (27). In the Fourier domain, *Z*_*m*_ is given by

(43) $$\begin{array}{l} \;Z_{m}\left(j\omega \right)=ℜ\left\{Z_{m}\left(j\omega \right)\right\}+jℑ\left\{Z_{m}\left(j\omega \right)\right\},\text{ with }\\ ℜ\left\{Z_{m}\left(j\omega \right)\right\}=R_{1}\cdot \frac{R_{2}\cdot \left(R_{1}+R_{2}\right)+\omega^{2}\cdot L^{2}}{{\left(R_{1}+R_{2}\right)}^{2}+\omega^{2}\cdot L^{2}},\text{ and } \end{array}$$

(44) $$\begin{array}{l} ℑ\left\{Z_{m}\left(j\omega \right)\right\}=\frac{\omega \cdot L\cdot R_{1}^{2}}{{\left(R_{1}+R_{2}\right)}^{2}+\omega^{2}\cdot L^{2}}. \end{array}$$

At any operating point *Q*_*m*_ along the entire NDR region of the device DC *I*_*m*_–*V*_*m*_ characteristic, *R*_2_ is strictly negative, while *R*_1_ + *R*_2_ is strictly positive. It follows that the real part ℜ{*Z*_*m*_(*jω*)} of the memristor local impedance goes negative within the angular frequency range

(45) $$\begin{array}{l} \omega_{l,Z_{m}}=-\frac{\sqrt{-R_{2}\cdot \left(R_{1}+R_{2}\right)}}{L}<\omega <\omega_{r,Z_{m}}\\ =+\frac{\sqrt{-R_{2}\cdot \left(R_{1}+R_{2}\right)}}{L} \end{array}$$

**Figure 16:**
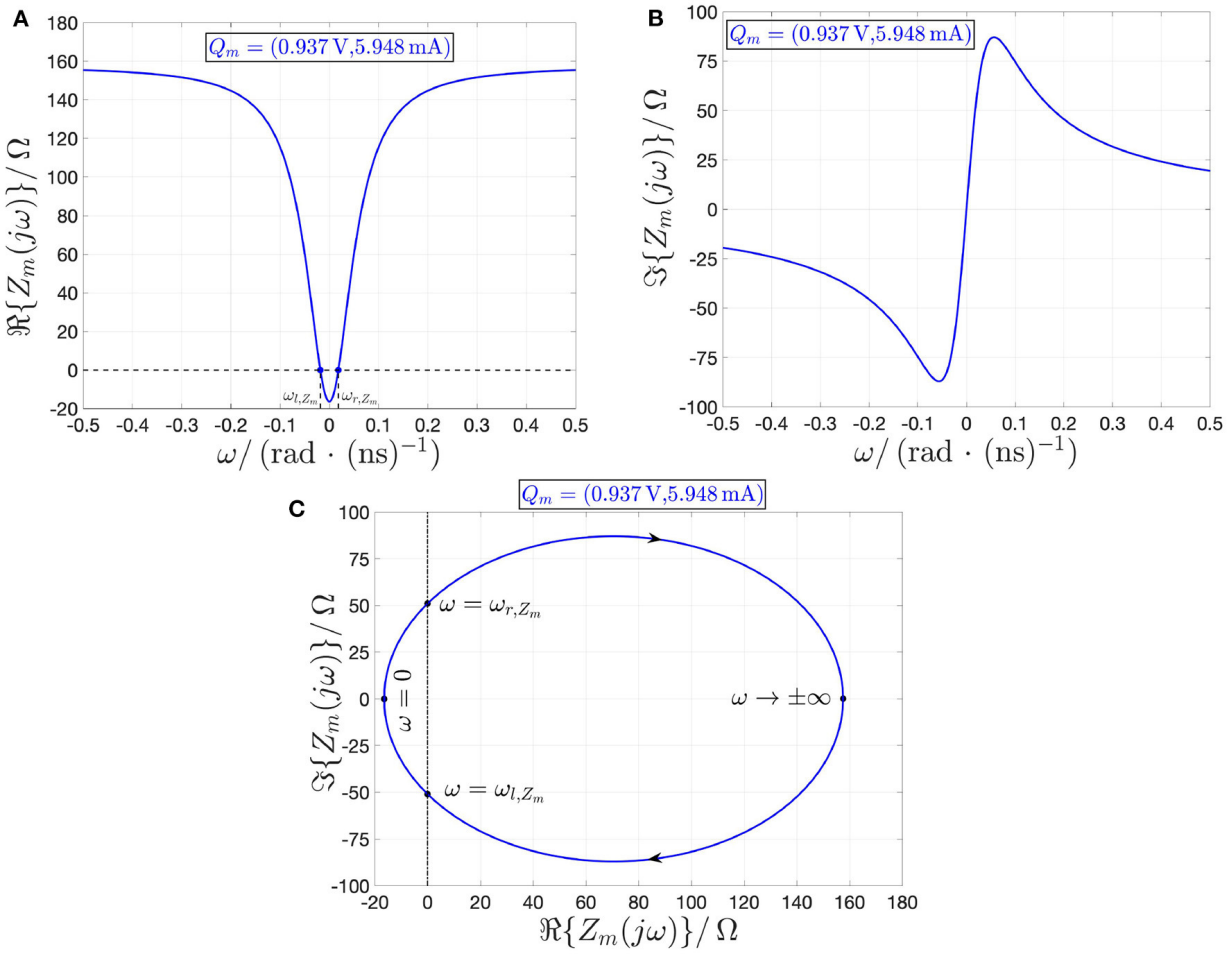
Loci of the real **(A)** and imaginary **(B)** parts of the local impedance *Z*_*m*_(*jω*) of the NbO memristor vs. the angular frequency ω at the memory state operating point *X* = 478, corresponding to the NDR current-voltage bias point *Q*_*m*_ = (*V*_*m*_, *I*_*m*_) = (0.937 V, 5.948 mA). **(C)** ℑ{*Z*_*m*_(*jω*)} vs. ℜ{*Z*_*m*_(*jω*)} locus, extracted from **(A,B)**, with arrows indicating the evolution of the trajectory point along the Nyquist plot as the angular frequency increases from −∞ to +∞. The real part of the local impedance is negative for ω ∈ (ω_*l*,*Z*_*m*__, ω_*r*,*Z*_*m*__), where ω_*r*,*Z*_*m*__ = −ω_*l*,*Z*_*m*__ = 18.305 Mrad· s^−1^. It follows that, under current control, the memristor is locally active and stable at the state operating point *X* = 478, and, as a result, it is said to poised on the EOC therein.

For example, Figures 16A,B illustrate the real and imaginary parts of the memristor local impedance *Z*_*m*_(*jω*) about the operating point *Q*_*m*_ = (*V*_*m*_, *I*_*m*_) = (0.937 V, 5.948 mA) as a function of the angular frequency ω, respectively. Here, ℜ{*Z*_*m*_(*jω*)} < 0 Ω for the angular frequency range specified in Equation (45), where ω_*r*,*Z*_*m*__ = −ω_*l*,*Z*_*m*__ = 18.305 Mrad· s^−1^, as indicated also in the corresponding Nyquist plot, illustrated in Figure 16C.

All in all, as reported in Table 2, under current control, the NaMLab NbO threshold switch is locally passive at each bias point on either of the two PDR branches of the DC current-voltage characteristic, while it is locally active and stable, i.e., it is poised on the EOC, throughout the NDR region of the *I*_*m*_–*V*_*m*_ locus.

## 4. Quasi-Static Behavior of the Micro-Scale Device

**Figure 17:**
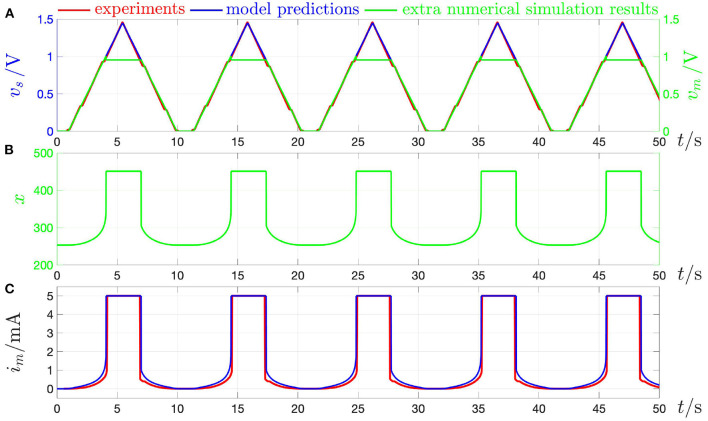
Current compliance-constrained response of the NbO memristor to a quasi-DC voltage stimulus. **(A)** Strictly positive triangular voltage waveform *v*_*s*_ of amplitude ${\hat{v}}_{s}=1.45$V and period *T* = 9 s falling directly across the device only when the latter is out of the compliance regime, and time waveform of the memristor voltage, which, when the current through the micro-structure attains the compliance level, descends abruptly to a fixed value, keeping unchanged thereafter till the time instant, at which the triangular stimulus descends below it. **(B)** Memory state response to the current-constrained quasi-DC voltage excitation test. **(C)** Current flowing through the device under the compliance-constrained quasi-static test. The red and blue waveforms in **(A,C)** were respectively extracted from experimental measurements performed at a chamber ambient temperature *T*_*amb*_ of −20°C and from a numerical simulation of the model, consisting of the DAE set (3) and (4), with state evolution and memductance functions, respectively, expressed by Equations (5) and (6), under nominal conditions, and of the DAE set (7) and (8), with state evolution and memristance functions, respectively, expressed by Equations (9) and (10), with *i*_*m*_ = *I*_*c*_ = 5 mA, in compliance mode, for an initial condition *x*_0_ ≜ *x*(0 s) equal to 253.15. The green waveforms in **(A,B)** report further results from the model simulation. Plotting the memristor current *i*_*m*_ from **(C)** against the source voltage *v*_*s*_ from **(A)**, i.e., against the voltage, which would fall across the micro-device in case it would never operate in compliance mode, results in the quasi-static current-voltage locus shown in Figure 19 through a blue dotted (magenta dashed) curve, as extracted via measurements (through a model numerical simulation). Six numbered arrows, referring to the magenta dashed curve, show the evolution of the trajectory point (*v*_*s*_, *i*_*m*_) over time in one input cycle. No point exists along the vertical line numbered 2 (5), when the micro-structure is abruptly transitioning into (out of) the compliance regime, when the locus visits data paths 3, and 4, i.e., the vertical lines are only drawn for visualizing better the sequence of events preceding and following the current-limited phase (see also Figure 20B on a compliance-free quasi-DC voltage excitation test with *R*_*s*_ = 0 Ω). With respect to the numerical simulation result, in case *i*_*m*_ from **(C)** were plotted against *v*_*m*_ from **(A)** in Figure 19, the resulting locus would differ from the *i*_*m*_–*v*_*s*_ characteristic only over the compliance phase, when the only trajectory point from the arrowed path 3 → 4 of the magenta dashed curve, which one would observe in this case after $Q_{m,A}^{\left(quasi-DC\right)}$ and before $Q_{m,C}^{\left(quasi-DC\right)}$ would be $Q_{m,B}^{\left(quasi-DC\right)}$, which coincides with the only possible device operating point under current control with *i*_*m*_ = *I*_*c*_, as may be inferred via the load line analysis ($Q_{m,B}^{\left(quasi-DC\right)}$ represents the unique intersection point between the device DC *I*_*m*_–*V*_*m*_ locus, shown in black in Figure 19, and the horizontal load line *I*_*m*_ = *I*_*c*_ associated to the compliance phase, when the quasi-DC voltage source across the threshold switch acts as a DC current source of value *I*_*s*_ = *I*_*c*_).

This section is devoted to explain the difference between the quasi-DC and the DC behaviors of the memristor device so as to clarify once and for all some of the misconceptions, which are often encountered in the literature in this regard. The response of the threshold switch to a slowly varying purely AC periodic stimulus, typically referred to as *quasi-static* or *quasi-DC* input, may either deviate from or accurately mimic the DC behavior depending upon the nature of excitation waveform, i.e., whether it is in voltage or in current form. Figure 17 shows both experimental results (in red) and model solutions (predictions of measurements in blue and further numerical results in green) on the NaMLab memristor dynamics in response to a strictly positive purely AC periodic quasi-static voltage stimulus of triangular shape, amplitude ${\hat{v}}_{s}=1.45\text{ V}$, and period *T* = 9 s^29^, as shown in plot (A), for *x*_0_ ≜ *x*(0 s) = 253.15, and under current constraint measures. In this regard, in order to prevent an irreversible damage to the memristor physical structure during the device abrupt off-to-on resistance switching process, the maximum current flowing through the micro-scale device, was limited to a compliance value *I*_*c*_ of 5 mA both in the lab and in the model numerical simulation. Figures 17A–C show the time evolution of memristor voltage *v*_*m*_, state *x*, and current *i*_*m*_ in this current-constrained quasi-DC voltage excitation test, respectively. Plotting *i*_*m*_ against *v*_*s*_ on the basis of the experimental measurements (model numerical simulations) results in the blue dotted (magenta dashed) quasi-static locus shown in Figure 19. Note that the locus of the memristor current vs. the memristor voltage coincides with the *i*_*m*_–*v*_*s*_ characteristic when the device is out of the compliance regime, i.e., as the trajectory point moves from^30^ $Q_{m,O}^{\left(quasi-DC\right)}$ to $Q_{m,A}^{\left(quasi-DC\right)}$, during the ascending phase of the voltage stimulus, and from $Q_{m,C}^{\left(quasi-DC\right)}$ to $Q_{m,O}^{\left(quasi-DC\right)}$, during the descending phase of the voltage stimulus, while it consists of a single point, namely $Q_{m,B}^{\left(quasi-DC\right)}$, which coincides with the intersection between the device DC *I*_*m*_–*V*_*m*_ characteristic and the horizontal load line *i*_*m*_ = *I*_*c*_, throughout the compliance phase (Slesazeck et al., 2014). The reason why the device quasi-DC *i*_*m*_–*v*_*m*_ locus does not visit NDR points between $Q_{m,A}^{\left(quasi-DC\right)}$ and $Q_{m,B}^{\left(quasi-DC\right)}$ along the device DC *I*_*m*_–*V*_*m*_ characteristic, also shown in black in the same figure, lies in the instability of these points under voltage control. Moreover, the load line-based analysis of the unique memristor operating point $Q_{m,B}^{\left(quasi-DC\right)}$ at compliance clarifies why, as may be inferred by inspecting plot (A) in Figure 17, the memristor voltage follows the 0.11 Hz-periodic triangular stimulus only as long as the micro-structure operates out of the compliance regime, while it abruptly decreases to the voltage of the only possible device bias point for *i*_*m*_ = *I*_*c*_, as soon as the device sets into the compliance mode, keeping unchanged afterwards, till the periodic voltage stimulus decreases below the constant memristor voltage level *V*_*m,B*_, i.e., the abscissa of $Q_{m,B}^{\left(quasi-DC\right)}$).

Remark 7. *Basically, the periodic voltage generator v*_*s*_, *appearing in parallel to the threshold switch, begins to source a DC current *I*_*s*_, equal to the earlier specified upper bound *I*_*c*_, as soon as the device current is about to exceed the compliance level during the abrupt turn-on process of the micro-structure, and keeps operating in this “DC current mode” as long as the periodic voltage waveform, which it would have sourced continuously in case no current limitation strategy were set into place, is about to decrease below the voltage of the only possible DC operating point, which the device may admit throughout the compliance phase. In order to reproduce the device behavior in compliance mode using our mathematical description, as the current flowing through the micro-structure attains the specified threshold *I*_*c*_, the voltage-controlled device DAE set (3) and (4), with state evolution function g*(·, ·) *expressed by Equation (5) and memductance function G*(·) *described via Equation (6), is recast as the model of a current-controlled generic memristor, namely (7) and (8), with state evolution function and memristance function, respectively, expressed by Equations (9) and (10), in which *i*_*m*_ is fixed to the compliance current*^31^ *I*_*c*_.

**Figure 18:**
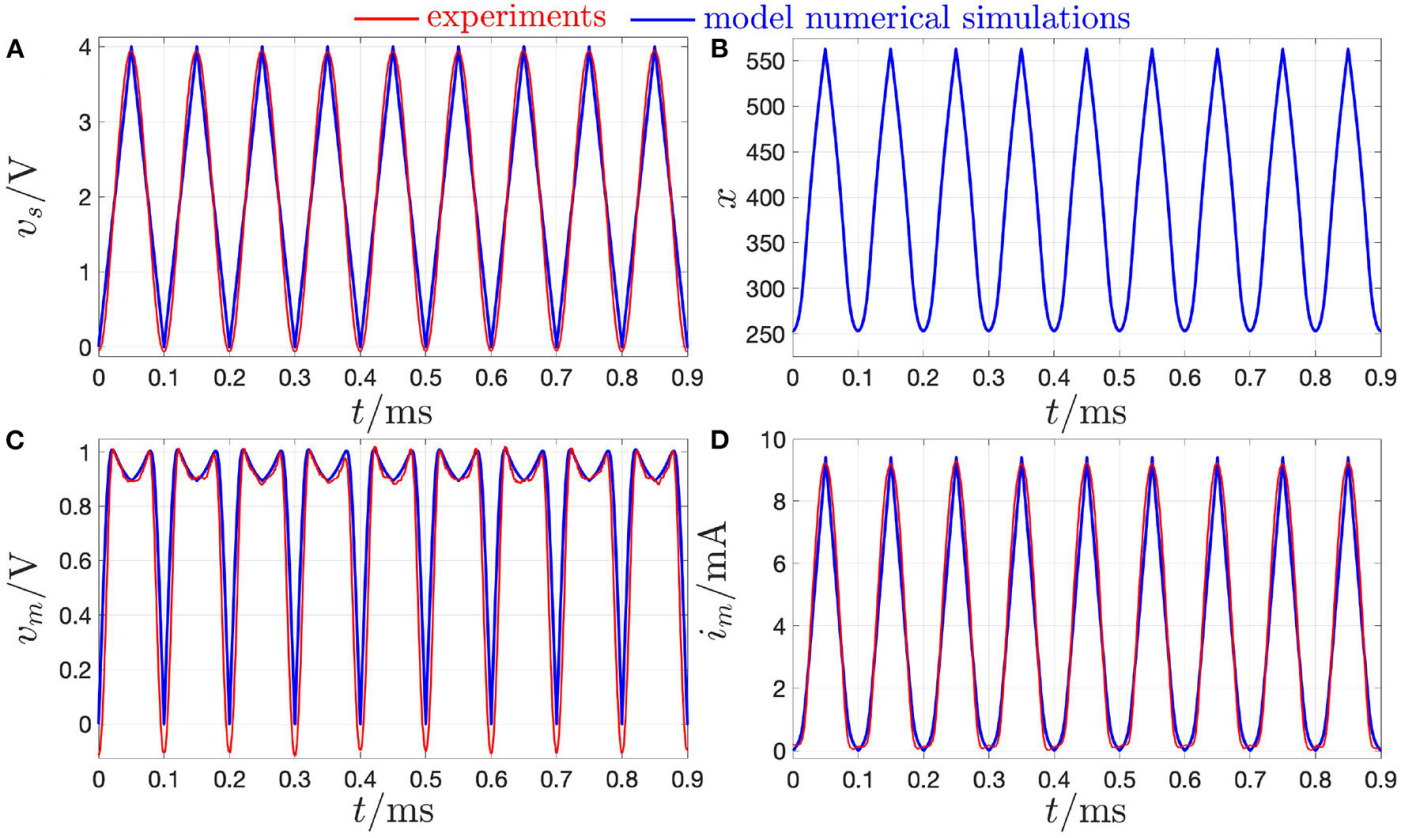
Response of the NbO memristor to the application of a purely AC voltage stimulus across the one-port composed of the series connection between the same memristor and a linear resistor of resistance *R*_*s*_ = 330 Ω, as shown in Figure 8a. **(A)** Strictly-positive triangular voltage waveform of amplitude ${\hat{v}}_{s}=4$V and period *T* = 0.1 ms. Here, the linear resistor limits the maximum current, which may ever flow through the NbO device, thus no compliance-related measure needs to be taken to prevent the physical breakdown of the memristor as it switches on. **(B)** Time evolution of the memristor state upon the application of the periodic voltage waveform in **(A)** across the resistor-NbO device series one-port. **(C)** Memristor voltage over time under the AC voltage stimulus in **(A)**. **(D)** Time waveform of the memristor current in response to the AC input in **(A)**. The red and blue waveforms in **(A,C,D)**, respectively, extracted from experimental measurements, with the chamber hosting the device kept at an ambient temperature *T*_*amb*_ of −20°C, and from a numerical simulation of the model DAE set (3) and (4), with state evolution and memductance functions expressed by Equations (5) and (6), respectively, and under the initial condition *x*_0_ = 253.15. Plotting the memristor current *i*_*m*_ from **(D)** against the memristor voltage *v*_*m*_ from **(C)**, the resulting AC *i*_*m*_–*v*_*m*_ locus, shown in Figure 19 in the green (yellow) color, as extracted through measurements (as derived from a numerical simulation of the model), matches rather well the threshold switch DC *I*_*m*_–*V*_*m*_ locus, drawn in black in the same figure, despite the stimulus frequency is well beyond 0 Hz. Given that the threshold switch behaves similarly as under DC excitation, the 10 kHz AC input voltage in **(A)** may be practically considered as a quasi-static stimulus as far as the dynamics of its memory state are concerned. The agreement between the device quasi-static and DC current-voltage loci in this scenario is expected from the analyses of sections 2.3 and 3.3, since the voltage-based quasi-DC stimulus is applied across the combined memristor and the resistance *R*_*s*_ is chosen so as to stabilize the entire NDR branch of the threshold switch DC current-voltage characteristic.

**Figure 19:**
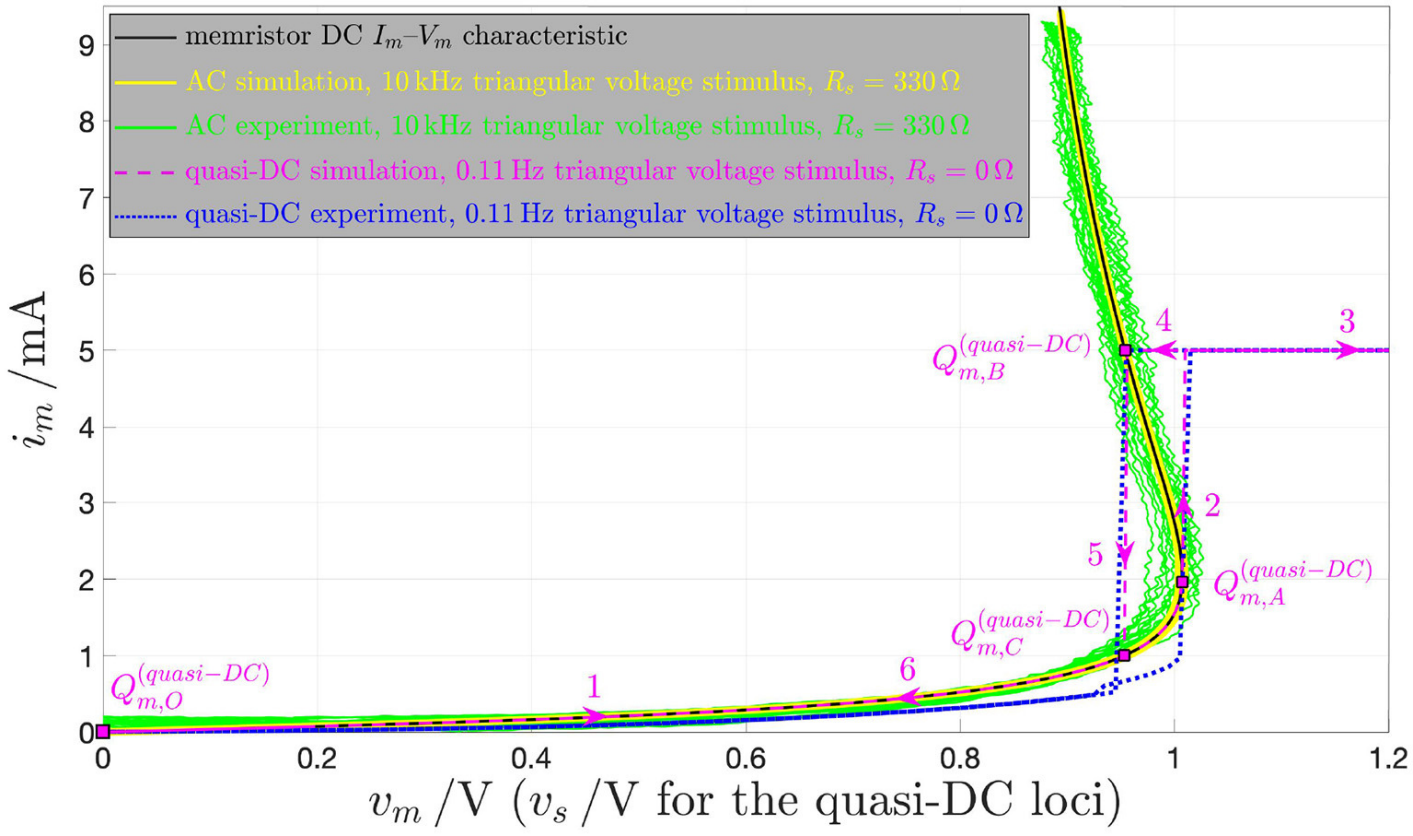
Black solid curve: DC *I*_*m*_–*V*_*m*_ locus of the NbO memristor, as derived from Figure 2C for *I*_*m*_ ∈ [0, 10] mA. Blue dotted (magenta dashed) curve: quasi-DC *i*_*m*_ vs. *v*_*s*_ characteristic of the NbO device, as extracted through measurements (by means of a numerical simulation of the model), where a purely positive periodic triangular voltage signal *v*_*s*_ of amplitude ${\hat{v}}_{s}=1.45$V and frequency *f* = 0.11 Hz was applied directly across the memristor—see Figure 17 for more details—until each time instant at which, during an abrupt off-to-on threshold switching process, the current *i*_*m*_ through the micro-structure attained a compliance level *I*_*c*_ set to 5 mA, while turning into a DC source of current *I*_*s*_ = *I*_*c*_ throughout each compliance mode phase. Note that, in accordance with similar current-limited quasi-DC loci reported in the literature, the horizontal axis for the blue dotted and magenta dashed characteristics shows the voltage, which would fall across the device in case it would never enter the compliance domain, i.e., in case it would always keep in the nominal operating mode, while, as explained in the text, the actual voltage dropping between the two memristor terminals under the current-limited regime is governed by the current-controlled Ohm law (8), with memristance *R* defined in Equation (10), and for *i*_*m*_ = *I*_*c*_. Referring to the numerical simulation result for this current-constrained quasi-DC voltage excitation test, plotting *i*_*m*_ vs. *v*_*m*_ would coincide with the magenta dashed *i*_*m*_–*v*_*s*_ locus out of compliance, i.e., from $Q_{m,O}^{\left(quasi-DC\right)}=\left(0\text{ V},0\text{ V}\right)$ to $Q_{m,A}^{\left(quasi-DC\right)}=\left(1.007\text{V},1.963\text{mA}\right)\approx Q_{m,1}=\left(1.007\text{ V},2.037\text{ mA}\right)$ (data path 1) in the ascending phase of the stimulus, and from $Q_{m,C}^{\left(quasi-DC\right)}=\left(0.953\text{ V},0.999\text{ mA}\right)$ back to $Q_{m,O}^{\left(quasi-DC\right)}$ in the descending phase of the stimulus (data path 6), while it would simply result in a single point, specifically $Q_{m,B}^{\left(quasi-DC\right)}=\left(0.954\text{ V},5\text{ mA}\right)$, throughout the compliance mode phase, instead of the data path 3 → 4 appearing when the horizontal axis shows the triangular excitation signal *v*_*s*_ (Slesazeck et al., 2014) (no data point exists along the vertical lines numbered 2 and 5, which simply show the abrupt device set and reset transitions, respectively). Green (yellow) solid curve: AC *i*_*m*_ vs. *v*_*m*_ characteristic of the NbO device extracted in a lab experiment (from a numerical simulation of the model), as the series combination between the memristor and a resistor with resistance *R*_*s*_ = 330 Ω is excited by a purely positive periodic triangular voltage signal *v*_*s*_ of amplitude ${\hat{v}}_{s}=4\text{ V}$ and frequency *f* = 10 kHz (see Figure 18 for details). As the experiment (model numerical simulation) reveals, the green (yellow) solid curve approximates with good accuracy the black-colored DC *I*_*m*_–*V*_*m*_ characteristic.

Now, if a series resistor of resistance *R*_*s*_, chosen in accordance with the inequality (15), in which the threshold value $\hat{r}$, defined in Equation (16), was found to be equal to 21.43 Ω at *X* = 411 for our threshold switch, is now inserted between a quasi-DC voltage stimulus and the memristor, the latter undergoes dynamics, which closely approximate its DC behavior. With reference to Figure 18, in which the blue and red color is, respectively, adopted to visualize the lab measurements and their model predictions, when a strictly positive purely AC periodic triangular voltage waveform^32^ *v*_*s*_ of amplitude ${\hat{v}}_{s}=4\text{ V}$ and period *T* = 0.1*ms*, as shown in plot (A), is applied to the series combination of a series resistor of resistance *R*_*s*_ = 330 Ω and the NamLab NbO memristor sample, the state, voltage, and current of the threshold switch exhibit the time waveforms illustrated in plots (B), (C), and (D), respectively. Here, where it is the series resistance which acts as memristor current limiter, plotting the memristor voltage against the memristor current—see the green (yellow) locus in Figure 19 in regard to the experimental result (the model numerical simulation)—replicates precisely the threshold switch DC *I*_*m*_–*V*_*m*_ characteristic, drawn in black in the same figure. The agreement between the quasi-static and DC behaviors of the voltage-controlled threshold switch in this case descends from the fact that the chosen value for the resistance *R*_*s*_ of the series resistor stabilizes the entire device DC locus NDR branch, which, on the other hand, was left unstable, except for the compliance-controlled operating point *Q*_*m,B*_, in the current-constrained quasi-DC voltage excitation test shown in Figure 17. In case the linear resistance *R*_*s*_, set in series to the threshold switch so as to stabilize one of its NDR operating points, according to constraint (13), is however not sufficiently large to satisfy condition (15), part of the NDR region of the device DC *I*_*m*_-*V*_*m*_ locus will keep unstable. Let us gain some insight into the effects of the partial stabilization of the device DC locus NDR branch on its response to a compliance-free quasi-static voltage stimulation.

**Figure 20:**
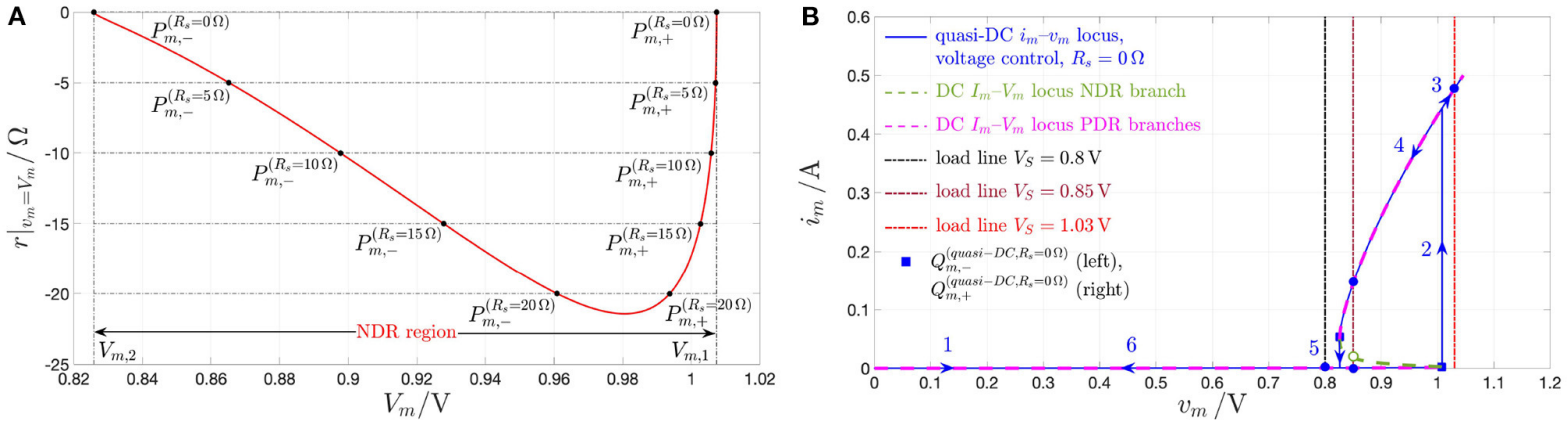
**(A)** Small-signal resistance *r*|_*v*_*m*_=*V*_*m*__ (in red) vs. memristor DC voltage *V*_*m*_ for all the possible NDR DC operating points of the threshold switch (refer also to Figures 2, 3). In general, for each admissible negative value $\tilde{r}$ of the small-signal resistance in the NDR region, the horizontal line $r=\tilde{r}$ crosses the *r*|_*v*_*m*_=*V*_*m*__ vs. *V*_*m*_ locus in two points; let us call them $P_{m,-}^{\left(R_{s}=-\tilde{r}\right)}$ and $P_{m,+}^{\left(R_{s}=-\tilde{r}\right)}$, enclosing the set of NDR operating points, which would not be stabilized by the insertion of a linear resistor of resistance $R_{s}=-\tilde{r}$ in series with the threshold switch (here $\tilde{r}$ is stepped across the range of values {0 Ω, −5 Ω, −10 Ω, −15 Ω, −20 Ω}). $P_{m,-}^{\left(R_{s}=0\Omega \right)}$ ($P_{m,+}^{\left(R_{s}=0\Omega \right)}$) lies in correspondence with the uppermost (lowermost) point of the NDR region of the device DC *I*_*m*_–*V*_*m*_ characteristic, identified via the memristor DC voltage *V*_*m*_ = *V*_*m*,2_ = 0.826 V (*V*_*m*_ = *V*_*m*,1_ = 1.007 V). The horizontal axis location of $P_{m,-}^{\left(R_{s}=5\Omega \right)}$ ($P_{m,+}^{\left(R_{s}=5\Omega \right)}$) is identified by the DC *V*_*m*_ value of 0.865 V (1.007 V). The abscissas of points $P_{m,-}^{\left(R_{s}=10\Omega \right)}$ and $P_{m,+}^{\left(R_{s}=10\Omega \right)}$, respectively, are 0.898 and 1.006 V. The memristor DC voltage at point $P_{m,-}^{\left(R_{s}=15\Omega \right)}$ ($P_{m,+}^{\left(R_{s}=15\Omega \right)}$) is 0.928 V (1.003 V). Finally, the *V*_*m*_ values of points $P_{m,-}^{\left(R_{s}=20\Omega \right)}$ and $P_{m,+}^{\left(R_{s}=20\Omega \right)}$ are in turn 0.961 and 0.994 V. **(B)** Device quasi-DC locus (in blue solid line style) emerging on the *v*_*m*_–*i*_*m*_ plane over one input cycle under compliance-free voltage stimulation of the sole device (*R*_*s*_ = 0 Ω) with a periodic triangular voltage signal of amplitude ${\hat{v}}_{s}=1.04$V and period *T* = 10 s, as follows from a numerical simulation of the proposed device model from an initial condition *x*_0_ of value 253.15. Six numbered arrows, superimposed on top of the blue solid curve, visualize the path, which the quasi-DC point (*v*_*m*_, *i*_*m*_) follows over time in one period. No data point exists on the vertical blue solid line numbered 2 (5), which simply shows the device abrupt set (reset) transition. The PDR branches (NDR branch) of the device DC current-voltage characteristic are (is) plotted in dashed line style and magenta (green) color. A triplet of load lines, in dashed-dotted line style and different colors, is also depicted to indicate, once again, that only their crossings with intrinsically stable PDR parts of the device DC *I*_*m*_–*V*_*m*_ characteristic appear also as points of the quasi-static *i*_*m*_–*v*_*m*_ locus in blue solid line style. As expected from the analysis in section 2(b), the quasi-static locus visits none of the DC operating points, lying on the green dashed NDR branch, since, under voltage control, the latter may not be stabilized without a series resistor. Given that the vertical lines numbered 2 and 5 are data free, the quasi-DC locus is in fact composed of two disconnected branches, which are separated by the entire NDR branch of the DC locus. Now, the uppermost (lowermost) quasi-DC point of the lower (upper) branch of the blue solid curve, indicated via a blue square marker, is located at $Q_{m,+}^{\left(quasi-DC,R_{s}=0\Omega \right)}=\left(1.007\text{ V},1.990\text{ mA}\right)$ [$Q_{m,-}^{\left(quasi-DC,R_{s}=0\Omega \right)}=\left(0.826\text{ V},53.399\text{ mA}\right)$], in good accordance with the *V*_*m*_ coordinate of $P_{m,+}^{\left(R_{s}=0\Omega \right)}$ ($P_{m,-}^{\left(R_{s}=0\Omega \right)}$) predicted in **(A)**.

**Figure 21:**
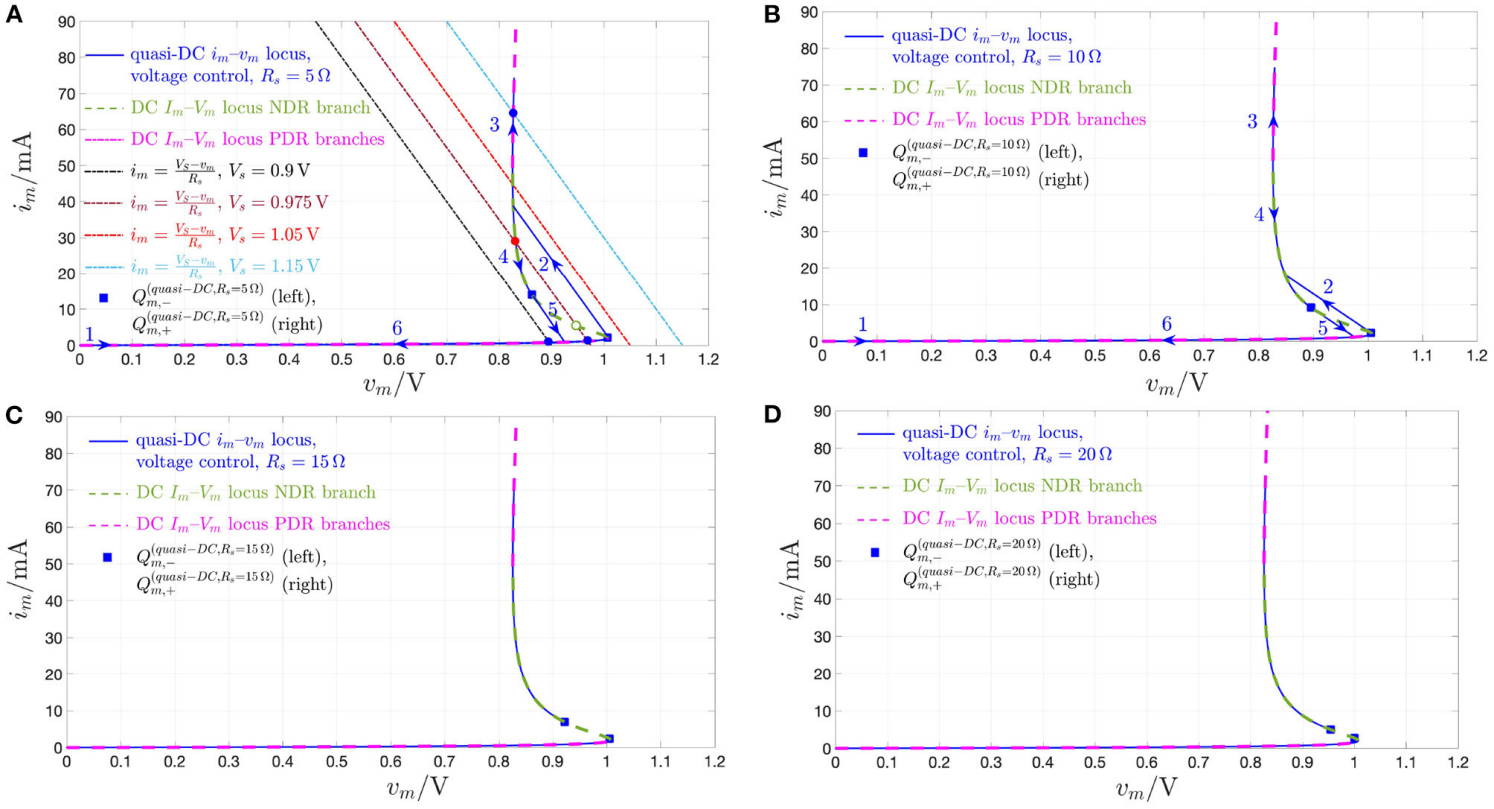
Blue solid curves: Memristor quasi-static current-voltage loci over one cycle of a period-*T* triangular voltage signal applied across the series combination between the NaMLab micro-structure and a series resistor of variable resistance *R*_*s*_ without any current limitation measure, as results from numerical simulations of the device polynomial model with initial condition *x*_0_ = 253.15. The input period *T* was fixed to 10 s, while, in order to achieve a similar range in quasi-DC memristor currents, the amplitude ${\hat{v}}_{s}$ of the voltage stimulus was appropriately increased as *R*_*s*_ was assigned larger values. **(A)** *R*_*s*_ = 5 Ω, ${\hat{v}}_{s}=1.2$V. **(B)** *R*_*s*_ = 10 Ω, ${\hat{v}}_{s}=1.575$V. **(C)** *R*_*s*_ = 15 Ω, ${\hat{v}}_{s}=1.925$V. **(D)** *R*_*s*_ = 20 Ω, ${\hat{v}}_{s}=2.25$V. The PDR branches (NDR branch) of the device DC current-voltage characteristic are (is) plotted in dashed line style and magenta (green) color. As studied in section 2(c), the larger is the value assigned to $R_{s}=-\tilde{r}$, with $\tilde{r}$ assuming values in {−5 Ω, −10 Ω, −15 Ω, −20 Ω}, the wider is the range of memristor NDR operating points, which are stabilized through the introduction of the series resistor, and, as a result, form part of the quasi-static *i*_*m*_–*v*_*m*_ characteristic [in other words, the horizontal distance between the black-filled markers, connected by the unstable part of the dashed green NDR branch, which may not be observed in the quasi-static test, reduces from **(A)** to **(D)**]. With reference to **(A,B)**, where the quasi-DC loci are endowed with six numbered arrows to show the time evolution of the trajectory point (*v*_*m*_, *i*_*m*_) over one input cycle, no data point exists on the solid blue line numbered 2 (5), which is added simply to indicate the threshold switch abrupt set (reset) transition. Importantly, the direction of either of the set and reset discontinuous transitions is the same as the load line slope $-R_{s}^{-1}$. This explains why the set and reset transitions in **(A,B)** are not vertically oriented, unlike their counterparts in **Figure 20B**. Set and reset transitions (and numbered arrows) are not shown in **(C,D)** in order to avoid clutter. Four load lines are also drawn in **(A)** to reveal, once more, that only their intersections with inherently stable PDR parts or stabilized NDR parts of the device DC *I*_*m*_–*V*_*m*_ characteristic appear also as points of the quasi-static *i*_*m*_–*v*_*m*_ locus in blue solid line style. The coordinates of the two quasi-static points, highlighted via blue filled squares, and sitting on the lowest and highest locations of the upper and lower branch of the blue solid locus, respectively, are reported below for each of the four plots: **(A)** $Q_{m,-}^{\left(quasi-DC,R_{s}=5\Omega \right)}=\left(0.863\text{ V},14\text{ mA}\right)$ and $Q_{m,+}^{\left(quasi-DC,R_{s}=5\Omega \right)}=\left(1.007\text{ V},2.114\text{ mA}\right)$. **(B)** $Q_{m,-}^{\left(quasi-DC,R_{s}=10\Omega \right)}=\left(0.896\text{ V},9.202\text{ mA}\right)$ and $Q_{m,+}^{\left(quasi-DC,R_{s}=10\Omega \right)}=\left(1.006\text{ V},2.338\text{ mA}\right)$. **(C)** $Q_{m,-}^{\left(quasi-DC,R_{s}=15\Omega \right)}=\left(0.922\text{ V},6.953\text{ mA}\right)$ ($Q_{m,+}^{\left(quasi-DC,R_{s}=15\Omega \right)}=\left(1.005\text{ V},2.401\text{ mA}\right)$). **(D)** $Q_{m,-}^{\left(quasi-DC,R_{s}=20\Omega \right)}=\left(0.954\text{ V},4.997\text{ mA}\right)$ and $Q_{m,+}^{\left(quasi-DC,R_{s}=20\Omega \right)}=\left(1\text{ V},2.736\text{ mA}\right)$. For each value of $\tilde{r}$ in the aforementioned set, the *V*_*m*_ coordinates of the quasi-DC points $Q_{m,-}^{\left(quasi-DC,R_{s}=-\tilde{r}\right)}$ and $Q_{m,+}^{\left(quasi-DC,R_{s}=-\tilde{r}\right)}$ are then in good agreement with the abscissas of the corresponding points $P_{m,-}^{\left(R_{s}=-\tilde{r}\right)}$ and $P_{m,+}^{\left(R_{s}=-\tilde{r}\right)}$, which are reported in the caption of **Figure 20**, respectively.

Remark 8. *Figure 20A shows how the small-signal resistance r*|_*v*_*m*_ = *V*_*m*__ *of the threshold switch changes with its DC voltage throughout the NDR region, where *V*_*m*_ assumes values in the range* (*V*_*m*,2_, *V*_*m*,1_) = (0.826, 1.007)*V, as discussed in the derivation of the DC *X*–*V*_*m*_, *X*–*I*_*m*_, and *I*_*m*_–*V*_*m*_ characteristics of the volatile memristor [refer to Figures 2A–C from section (2)(b), respectively]. Importantly, in case the series resistance *R*_*s*_ is set to a certain value lower than* $\left|\hat{r}\right|$, *which, as specified in Equation (16), is equal to* 21.43 Ω *at V*_*m*_ = 0.981 *V, the graph in Figure 20A may be interrogated to derive the range of memristor DC voltage values, at which the operating points of the NbO micro-memristor keeps unstable. For example, with *R*_*s*_, respectively equal to* 0, 5, 10, 15, *and* 20 Ω, *the open set of unstable NDR V*_*m*_ *values is in turn* (0.826, 1.007) *V, covering the entire NDR region of the device DC locus*, (0.865, 1.007), (0.898, 1.006), (0.928, 1.003), *and* (0.961, 0.994) *V. Employing a numerical simulation of the polynomial model, Figure 20B shows via a blue solid curve the quasi-DC locus appearing over each input cycle on the *v*_*m*_–*i*_*m*_ plane upon the application of a periodic triangular voltage signal *v*_*s*_ of amplitude* ${\hat{v}}_{s}=1.1$ *V and period T* = 10 *s across the sole device (R*_*s*_ = 0 Ω*) without setting up any current upsurge control strategy over the course of the device turn-on process. The blue solid curve, endowed with numbered arrows indicating the time evolution of the trajectory point* (*v*_*m*_, *i*_*m*_) *over one input cycle, visits the device DC locus PDR branches (in magenta dashed line style), but, as expected from the discussion in section 2(b) as well as from the analysis of plot (A) in Figure 20, does not go through any operating point lying on the device DC locus NDR branch (in green dashed line style). The abscissa of the lowermost (uppermost) blue-filled square marker on the upper (lower) branch of the discontinuous*^33^ *quasi-DC i*_*m*_–*v*_*m*_ *locus is in fact* 0.826 *V* (1.007 *V*). *Finally, Figure 21 gives evidence for the decrease in horizontal distance between the blue square markers in the discontinuous locus appearing on the *v*_*m*_–*i*_*m*_ plane upon the application of the same compliance-free quasi-static voltage excitation as established for the simulation shown in Figure 20B through the series combination between the NaMLab memristor and a linear resistor *R*_*s*_, as the resistance of the latter is increased stepwise across the range* {5 Ω *plot (A),* 10 Ω, *plot (B),* 15 Ω, *plot (C),* 20 Ω *plot (D)*}. *This was expected from the analytical treatment of section 2(c), as well as from the investigation of plot (A) in Figure 20. In fact, the abscissas of the lowermost (uppermost) blue-filled square markers on the upper (lower) branches of the discontinuous*^34^ *quasi-DC i*_*m*_–*v*_*m*_ *loci in plots (A–D), respectively, are* 0.863 *V (*1.007 *V),* 0.896 *V (*1.006 *V),* 0.922 *V (*1.005 *V), and* 0.954 *V (*1 *V)*.

**Figure 22:**
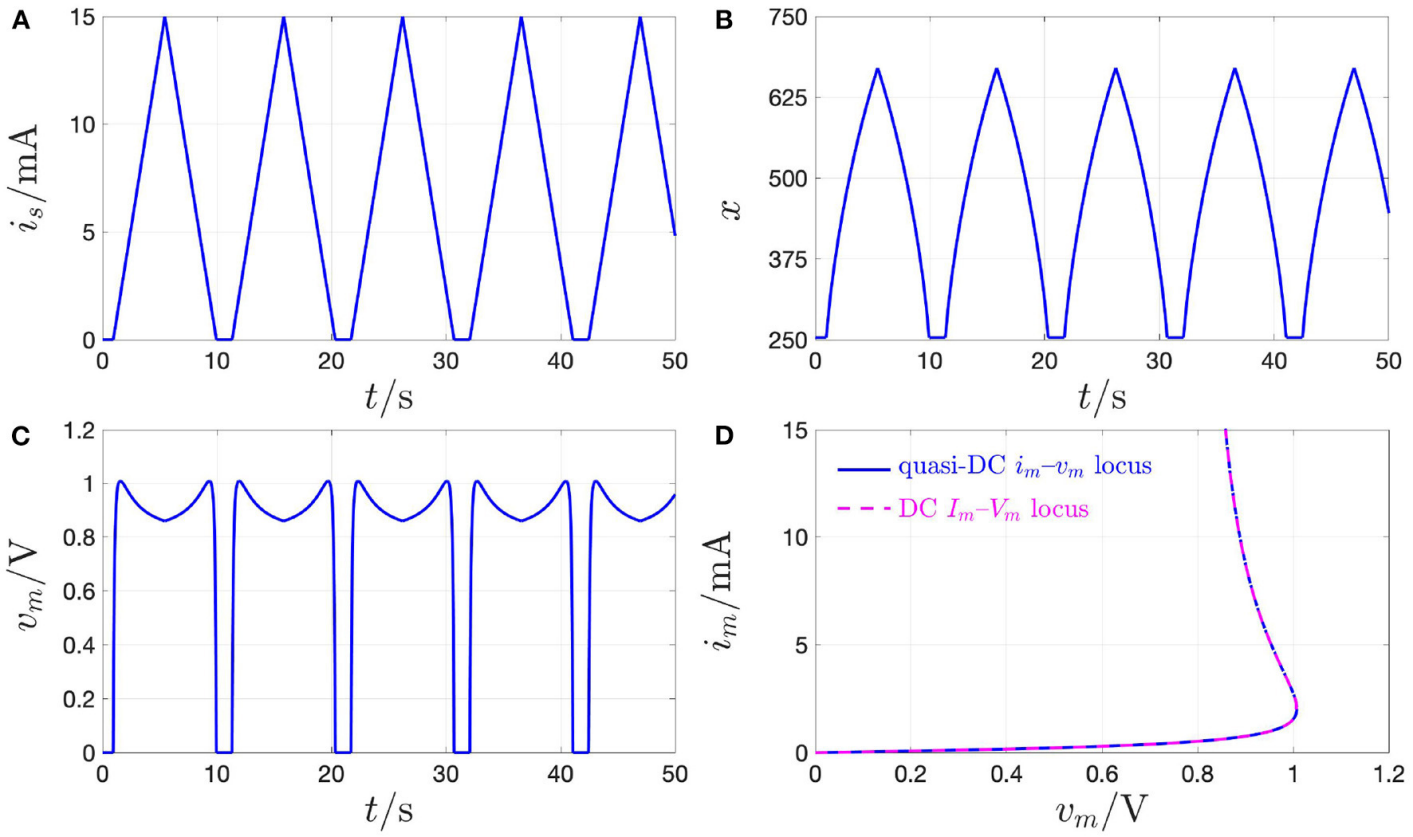
Response of the micro-scale device to a quasi-static stimulation in current form. **(A)** Time waveform of a strictly positive purely AC slowly varying periodic triangular current signal *i*_*s*_, of amplitude ${\hat{i}}_{s}=16\text{ mA}$ and period *T* = 9, *s*, inserted through the NbO threshold switch. **(B)** Time evolution of the memristor state in response to the quasi-DC stimulus in **(A)** from an initial condition *x*_0_ set to 253.15. **(C)** Voltage falling across the memristor over time, as it follows from the quasi-static current excitation of the device. **(D)** Blue curve: Memristor AC current *i*_*m*_-voltage *v*_*m*_ locus observed in the quasi-static test. Its qualitative and quantitative agreement with the device DC *I*_*m*_-*V*_*m*_ characteristic, shown in magenta here, was expected from the study of section 2.3, since the whole NDR region of the memristor DC locus is stable under current control.

Last but not least, inserting a quasi-static current through the memristor, the resulting AC *i*_*m*_ vs. *v*_*m*_ locus is found to resemble closely the device DC *I*_*m*_–*V*_*m*_ characteristic. This is shown by means of a numerical simulation of the DAE set (7) and (8), with state evolution and memristance functions, respectively, expressed by (9) and (10) and for *x*_0_ = 253.15. As illustrated in Figure 22A, a quasi-DC strictly positive AC periodic triangular current *i*_*s*_, of amplitude 15 mA and period *T* = 9 s, is let flow through the NbO memristor. It follows that the state and voltage of the device oscillate over time as depicted in Figures 22B,C, respectively. The resulting memristor AC current vs. voltage locus, highlighted with the blue color in plot (Figure 22D), approximates accurately the device *I*_*m*_–*V*_*m*_ characteristic, reproduced in magenta in the same figure. This was expected from the investigation of section 2.2, since the entire NDR branch of the memristor DC locus is stable under current control.

**Figure 23:**
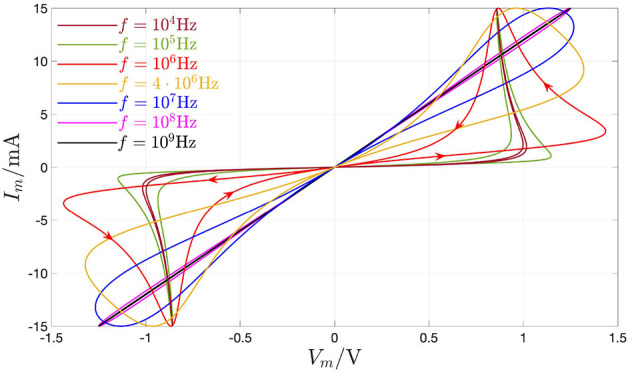
Pinched hysteresis loops (Chua, 2014) of tangential type (Biolek et al., 2011), emerging at steady-state on the *v*_*m*_–*i*_*m*_ plane under the application of an AC source, generating a sine wave current of the form *i*_*s*_ = $\hat{i}$_*s*_ · sin(2 · π · *f* · *t*) directly across the NbO memristor, for $\hat{i}$_*s*_ = 15 mA, as the frequency is stepped across the set of values {10^4^, 10^5^, 10^6^, 4 · 10^6^, 10^7^, 10^8^, 10^9^} Hz. These results were obtained by numerical simulation of the proposed polynomial-based DAE set (3) and (4), with *g*(*x, v*_*m*_) and *G*(*x*) expressed by Equations (5) and (6), respectively, and for an initial condition fixed to *x*_0_ = 253.15. For the scenario associated to the lowest input frequency in the aforementioned set, the locus resembles closely a quasi-static characteristic under current control, similarly as the plot of *i*_*m*_ vs. *v*_*m*_ in Figure 22D, with the only difference that here the input assumes also negative values. As the frequency is increased, first the overall loop lobe area is found to increase, while, concurrently, the shape of the characteristic differentiates itself more and more from the DC *I*_*m*_ vs. *V*_*m*_ locus (arrows are superimposed on top of one of the characteristics, specifically the red one observed for *f* = 10^6^ Hz, to provide evidence for the tangential nature of the loop). Then, after a certain threshold frequency, as expected of all memristors, the pinched hysteresis loop begins to shrink monotonously with the frequency (Chua, 2014). For very high frequencies the locus reduces to a straight line, as expected of all generic memristors (note that, differently from what happens for ideal and ideal generic memristors, the slope of the linear *i*_*m*_–*v*_*m*_ characteristic in this limiting cases depends upon the input amplitude) (Chua, 2015).

Before summing up the main results of this pedagogical article, it is worth to point out that the NbO micro-memristor exhibits the fingerprint of all memristors, namely *the pinched hysteresis loop* (Chua, 2014) on the voltage-current plane. Figure 23, based upon numerical simulations of the proposed polynomial-based model, depicts how the steady-state *i*_*m*_ vs. *v*_*m*_ locus evolves upon exciting the device with an AC source of the form *i*_*s*_ = $\hat{i}$_*s*_ · sin(2 · π · *f* · *t*) with $\hat{i}$_*s*_ = 15 mA, as the frequency is swept stepwise across the set of values {10^4^, 10^5^, 10^6^, 4 · 10^6^, 10^7^, 10^8^, 10^9^} Hz. Interestingly, each loop is tangential in the origin (Biolek et al., 2011), as highlighted for a representative scenario, specifically for *f* = 10^6^ Hz, by showing the direction of motion of a trajectory point along the associated characteristic over time (see the sequence of arrows on the red locus). For the lowest frequency from the aforementioned set, the locus is similar to a quasi-static characteristic under bipolar current control (compare it with the one in Figure 22D, where the current stimulus is strictly positive). As the input frequency is increased, the pinched hysteresis loop opens up and differentiates more and more from the DC locus. After a certain point, however, the loop lobe area begins to decrease progressively, as expected of all resistance switching memories (Chua, 2014), until, for the largest frequency value, the locus reduces to a straight line, as is the case for all generic memristors (Chua, 2015).

## 5. Discussion

Miniaturized memristor devices based upon certain materials, such as niobium dioxide, are able to amplify infinitesimal fluctuations in energy, and, when they exhibit such an impressive and peculiar capability, they are said to operate in the locally active regime. Since the potassium and sodium ion channels in biological axon membranes (Hodgkin and Huxley, 1952) are locally active memristors (Chua et al., 2012), which provide an essential contribution for the emergence of the all-or-none neuronal spiking behavior, it is clear that solid-state resistance switching memories, which admit a negative small-signal resistance over a range of operating points, which, as explained in this manuscript, constitutes a signature for their capability to enter the LA domain, will play a major role for the development of basic electronic building blocks of novel bio-mimetic neuromorphic networks. There have already been a few promising attempts to adopt these kinds of memristor physical realizations (Pickett and Williams, 2012) for the electronic implementation of biological neurons, the so-called neuronal memristors, or neuristors for short (Pickett et al., 2013; Yi et al., 2018), which have been further combined through various coupling arrangements to form memristive cellular automata endowed with computational universality (Pickett and Williams, 2013), and memristive cellular neural networks (M-CNNs), which, supporting the emergence of dynamic patterns (Weiher et al., 2019; Demirkol et al., 2021), may be employed to solve complex non-deterministic polynomial-time (NP)-hard optimization problems, such as the challenging task of coloring the vertices of an undirected graph (Weiher et al., 2021). In this article, we employed powerful techniques from non-linear circuit (Chua, 1987) and system (Ascoli et al., 2019; Corinto et al., 2020) theory as well as rigorous concepts from the theory of complexity to gain a thorough understanding of the non-linear dynamics of a locally active memristive microstructure from NaMLab. Given the inherent instability of the NDR branch of the device DC current *I*_*m*_-voltage *V*_*m*_ characteristic under voltage control, the condition for the stabilization of any operating point *Q*_*m*_ = (*V*_*m*_, *I*_*m*_) along it has been discussed from different viewpoints: (1) using a circuit-theoretic approach with the load line method; (2) applying a system-theoretic graphical tool, enabling the analysis of first-order systems, and known as DRM (Chua, 2018); (3) pursuing a classical linearization analysis of the memristor state equation in the time (frequency) domain on the basis of the eigenvalue (the pole of the device local admittance) about the operating point. The application of the LA theorem (Chua, 2005) to the device small-signal circuit model, extracted from the expression of the memristor local admittance, has subsequently allowed us to tabulate all the possible device operating modes, specifically the locally passive, the locally active and unstable, and the locally passive and stable regimes, the latter being typically referred to as EOC domain, depending upon its bias point as well as upon the nature of the control signal, being in voltage or current form. The availability of a full picture of the memristor behavior, derived by means of a rigorous theoretical analysis corroborated by experimental measurements on device samples, is instrumental for the future development of a systematic approach to design bio-inspired oscillatory networks. Finally, it is worth pointing out that this research provides clear evidence for the significant role that non-linear circuit and system theory assumes for gaining a deep insight into the operating principles of inherently non-linear memristive devices and circuits, which constitutes a crucial prerequisite for the development of a rigorous technique, aware of all the key system parameters in action, to design variability-tolerant neuromorphic electronic hardware.

## 6. Conclusions

In order for complex phenomena, e.g., the generation of action potentials in neuronal axon membranes, to emerge in biological systems, some of their essential units have to display the capability to enter a locally active operating mode, where they would be able to amplify the small-signal, upon the provision of a suitable DC energy source (Ascoli et al., 2020a). For example, the basic unit in the neuronal axon membrane may operate in the LA domain, and, most remarkably, in its “pearl” subdomain, referred to as EOC, and hosting the germ of complexity. This is possible because the membrane accommodates two voltage-controlled volatile memristors, namely the potassium and sodium ion channels^35^, which may boost local fluctuations in energy when biased on the NDR regions of their DC current-voltage loci (Chua et al., 2012). Recently, several research groups have presented solid-state volatile memory devices with DC characteristics including well-defined NDR regions (Pickett and Williams, 2012). Biasing any of such devices in some point, where the respective DC current-voltage locus features a negative slope, the memristor is said to operate in the LA regime. Superimposing a small-signal on top of the DC operating point, the locally active memristor may operate similarly as a MOS transistor biased in the saturation region, amplifying the local fluctuations at the expense of some DC power supply. Besides opening up the opportunity to design transistor-less small-signal amplification circuits as well as oscillatory networks, in which the emergence of spatiotemporal phenomena (Weiher et al., 2019) may be exploited to solve complex optimization problems (Weiher et al., 2021), the adoption of locally active memristors in electronics may also allow an accurate reproduction of the non-linear dynamics of potassium and sodium ion channels in innovative neuromorphic hardware (Pickett et al., 2013). In this article, we gained a deep insight into the non-linear dynamics of a NbO micro-scale memristor exhibiting an NDR region along the DC characteristic. The application of powerful techniques from non-linear circuit and system theory (Chua, 1987), and the adoption of methods from the theory of local activity (Chua, 2005), allowed us to understand the mechanisms behind the operation of this voltage-controlled volatile memristor from NaMLab in the LA regime to classify all the admissible operating modes of the threshold switch, and to clarify, once and for all, under which conditions the response of the micro-scale device to quasi-static stimuli matches its behavior under DC stress. Importantly, experimental measurements on a device sample fabricated at the premises of NaMLab GgmbH confirm the theoretical results, providing further support to the significance of this research contribution, which sheds light on the invaluable role that non-linear system theory plays in understanding the rich non-linear dynamics of memristors.

## Data Availability Statement

The raw data supporting the conclusions of this article will be made available by the authors, without undue reservation.

## Author Contributions

AA has conceived the main idea of the paper and written the entire manuscript. AA and SS have developed the device model. AA and SS performed the experimental measurements. AA and ASD have derived the device small-signal model, and applied the LA theory to the resulting circuit representation. RT, TM, and LC have provided a precious guidance throughout the development of the research work. All authors contributed to the article and approved the submitted version.

## Conflict of Interest

The authors declare that the research was conducted in the absence of any commercial or financial relationships that could be construed as a potential conflict of interest.

## Figures and Tables

**Figure 24:**
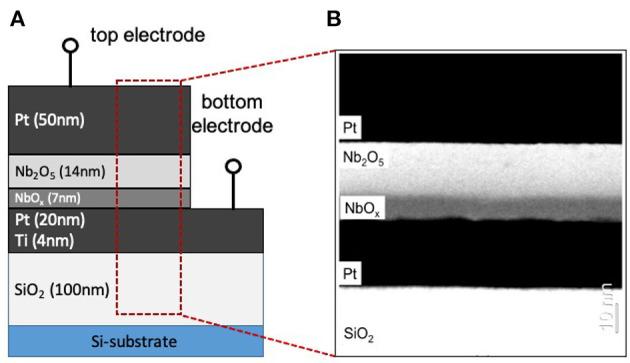
Schematics **(A)** and TEM image of the micro-device stack **(B)**.

## Footnotes

^1^A novel interesting perspective to interpret concepts from the LA theory has been recently provided in Garay (2017).

^2^The literature holds a number of impressive works, in which methods from the LA theory have been adopted to explain complexity in systems from disparate fields, including biology (Dogaru and Chua, 1998) and electronics (Min and Chen, 2004).

^3^An Appendix at the end of the paper provides a brief overview on the fabrication, material composition, geometric structure, and physics of the threshold switch under study.

^4^A circuit element is said to be *time invariant* if its input–output behavior does not change over time.

^5^The model of a current-controlled extended memristor may be derived *mutatis mutandis* from Equations (1) and (2) by invoking the duality principle (Chua, 1987).

^6^The memristor voltage *v*_*m*_ (current *i*_*m*_) is also referred to as the input or control (output) variable of a voltage-controlled memristor. By the duality principle (Chua, 1987), a similar nomenclature may be adopted *mutatis mutandis* for a current-controlled memristor.

^7^The memory state vector **x** of a volatile memristor is unable to store information, not even in digital binary form, for zero input. Therefore, precisely, it should be interpreted as a *volatile memory state*.

^8^If the constraint *G*(**x**, 0) ≠ ∞ were not included in the definition of an extended memristor, the DAE set (1) and (2) could be used to model other dynamic circuit elements, e.g., linear capacitors and inductors (Chua, 2014).

^9^Invoking the duality principle (Chua, 1987) a similar recipe may be written down *mutatis mutandis* for the determination of the DC voltage-current characteristic of a first-order current-controlled extended memristor.

^10^Referring to a first-order voltage-controlled extended memristor, the *Unfolding Theorem* (Chua, 2011) envisages the expansion of the state evolution function *g*(**x**, *v*_*m*_) in Equation (1) and of the memductance function *G*(**x**, *v*_*m*_) in Equation (2), for **x** = *x*, into sums and products of polynomial series in input and state variables.

^11^Keeping the maximum current flowing through the device below an upper bound of about 15 mA, the threshold switching dynamics emerging in the NbO micro-film are not found to be accompanied by non-volatile memory effects. However, if higher currents are let flow through the device, the latter is found to feature distinct resistive levels for no input (Mähne et al., 2013) besides maintaining an abrupt switching behavior upon excitation. For modeling the NbO memristor accurately under these circumstances, the dynamic law of another non-volatile memory state needs to be specified (Slesazeck et al., 2016). Since the device was operated in the low current regime during all the experimental investigations associated to this research, a volatile memristor model is adopted in this manuscript to capture the dynamics of the NbO threshold switch. However, for pedagogical reasons, and for the sake of completeness, some of the theoretical results, descending from the analysis of this model, e.g., the device DC current–voltage characteristic, shall cover also the high current regime.

^12^*V*_*m*_, *X*, and *I*_*m*_ denote the DC values for memristor voltage, state, and current, respectively. The triplet (*V*_*m*_, *X, I*_*m*_) defines a DC operating point for the memristor.

^13^Setting to 0 the state evolution function (5) for *V*_*m*_ = *V*_*m,a*_ = 0 V, the formula for the unique memristor state equilibrium is easily found to be *X*_*a*_ = *X*(*V*_*m,a*_) = *X*(*V*_*m*_ = 0 V) = −*a*_0_/*a*_1_ = 253.15.

^14^In this article, the abscissa and ordinate of a generic operating point *Q*_*m,i*_ – *i* ∈ {*I, II*, …}– along the device DC *I*_*m*_–*V*_*m*_ characteristic are denoted as *V*_*m,i*_ and *I*_*m,i*_, respectively.

^15^This approach is simpler than the one assuming a voltage control, since, here, the “dual” recipe of the procedure illustrated in Figure 1, applicable to any current-driven extended memristor model, would require the numerical calculation of a single zero for the state evolution function (9) for each DC value *I*_*m*_ assigned to the memristor current *i*_*m*_ (as may be inferred from Figure 2B, *X* is a single-valued function of *I*_*m*_). Importantly, under current sweep, each memristor state DC operating point *X* is found to be *globally asymptotically stable*, as will be discussed in more depth shortly (see Figure 2B for more details). As a result, according to the color coding convention adopted in Figure 2, all the points in the loci from plots (A–C) should be marked in blue under current control.

^16^In Figure 12, the lower (upper) PDR branch of the device DC *I*_*m*_–*V*_*m*_ locus will be referred to as PDR region 1 (2).

^17^Let a first-order dynamical system be described by a non-linear ODE of the form *ẋ* = *f*(*x*, μ), where *x* ∈ ℝ denotes the state variable, μ ∈ ℝ stands for a control parameter, and *f*(·, ·) : ℝ × ℝ → ℝ defines the state evolution function. Plotting the time derivative *ẋ* of the state variable vs. the state variable itself for a specific value $\bar{\mu }$ of the control parameters μ, and endowing the resulting locus with arrows pointing toward the east (west) on the positive (negative) half plane, provides a *state dynamic route* (SDR), illustrating the path, which *x* follows from any initial condition *x*_0_ ≜ *x*(0) in this scenario. An ODE equilibrium $\bar{x}$ for $\mu =\bar{\mu }$ corresponds to a state, which lies along the horizontal axis on the $f\left(x,\bar{\mu }\right)$ vs. *x* locus (*ẋ* vanishes at its location), and is said to be asymptotically stable (to be unstable) if the SDR features a negative (positive) slope therein, implying that, perturbing the system slightly away from its position, the system solution moves back to (departs from) it as time proceeds. The *dynamic route map* (DRM) of the first-order system is a family of SDRs, one for each of the admissible values of the control parameter μ. Importantly, *X* is the symbol adopted in this article to denote a zero of the memristor state evolution function in Equations (5) and (9), namely *g*(*x, v*_*m*_) (*f*(*x, i*_*m*_) = *g*(*x, R*(*x*) · *i*_*m*_), with *R*(*x*) = *G*^−1^(*x*), as established by Equation (10), and *G*(*x*) expressed by formula (6)) under a given DC *V*_*m*_ (*I*_*m*_) value. *X*, referred to as an *equilibrium* for the memristor state Equations (3) and (7), with state evolution function expressed by formula (5) and (9), under the specified bias voltage (current) stimulus *V*_*m*_ (*I*_*m*_), indicates a possible *DC operating point* for the memristor state in this scenario. Finally, it is worth mentioning that, recently, the DRM graphical tool has been extended to nonlinear dynamical systems with two degrees of freedom (Tetzlaff et al., 2020), which shall allow, for example, an in-depth study of the nonlinear dynamics of second-order memristors.

^18^Note that a unique memristor state *X* is associated to each point *Q*_*m*_ = (*V*_*m*_, *I*_*m*_) along the DC *I*_*m*_–*V*_*m*_ characteristic, as clear from Figure 2.

^19^Inspecting the mathematical formula (11) for the voltage *v*_*m*_ falling across the memristor in the circuit shown in Figure 8A for *R*_*s*_ ≠ 0 Ω, it is clear that the DC value *V*_*s*_ of the voltage source, which allows the load line, described by Equation (12), to cross the device DC current-voltage locus in the desired NDR operating point *Q*_*m*_ depends upon the choice of the resistance *R*_*s*_.

^20^Invoking the duality principle (Chua, 1987), similar conclusions apply *mutatis mutandis* to a first-order current-controlled two-terminal memristor. Furthermore, the local activity definition may be extended also to higher-order one- and multi-ports of memristive or non-memristive nature, as explained in Chua (1998).

^21^According to this convention, once a reference direction is chosen for the voltage falling across a two-terminal electrical circuit element, the current is assumed to flow into the one-port from the terminal, which is assigned a positive polarity (Chua, 1987).

^22^Invoking the duality principle (Chua, 1987), similar conclusions apply *mutatis mutandis* to a first-order current-controlled two-terminal memristor. Furthermore, the LA theorem may be generalized for the analysis of higher-order one- and multi-ports of memristive or non-memristive nature, as described in Chua (1998).

^23^For a first-order current-controlled memristive one-port, the local transfer function about *Q*_*m*_ is defined as *H*_*m*_(*s*) ≜ *Z*_*m*_(*s*), where $Z_{m}\left(s\right)≜\frac{L\left(\delta v_{m}\left(t\right)\right)}{L\left\{\delta i_{m}\left(t\right)\right\}}$ represents the *device small-signal impedance*.

^24^With reference to the 4th condition, $Y_{m}^{*}\left(j\omega \right)$ denotes the complex conjugate of *Y*_*m*_(*jω*).

^25^The residue of a *mth*-order pole *s* = *s*_*p*,*Y*_*m*__ of the device local admittance *Y*_*m*_(*s*) may be calculated via $k_{s_{p,Y_{m}}}=\frac{1}{\left(m-1\right)!}\cdot \underset{s\to s_{p,Y_{m}}}{\lim}\frac{d^{m-1}}{ds^{m-1}}\left({\left(s-s_{p,Y_{m}}\right)}^{m}\cdot Y_{m}\left(s\right)\right)$.

^26^The 3rd condition may never hold true for a first-order one-port.

^27^Unless otherwise stated, in the remainder of the paper the memristor small-signal admittance *Y*_*m*_ will be expressed in terms of the parameters of the device small-signal equivalent circuit model of Figure 8B, i.e., through Equation (27).

^28^By device instantaneous resistance we intend here its memristance.

^29^Given that, as anticipated earlier, it is reasonable to associate the device memory state *x* to its internal temperature *T* expressed in units *K*, the initial condition *x*_0_ = 253.15 corresponds to $T_{0}≜T\left(0\text{ s}\right)=-20{}^{\circ}$, which coincides with the ambient temperature *T*_amb_ set in the chamber hosting the threshold switch during the lab test.

^30^The superscript in the symbol of a generic point $Q_{m,i}^{\left(quasi-DC\right)}$ – *i* ∈ {*I, II*, …}–along a device quasi-DC *i*_*m*_–*v*_*m*_ locus is meant to highlight the quasi-static nature of the underlying characterization test, especially for the purpose of comparing the threshold switch response to approximately static excitations to its behavior under DC stress. In the remainder of the paper, a generic point of this kind is referred to as *quasi-DC point* so as to differentiate its name from the one— specifically DC operating or bias point—attributed to a generic point *Q*_*m,i*_ along the device DC *I*_*m*_–*V*_*m*_ characteristic.

^31^With reference to Figure 17, due to the extremely fast dynamics of the device during on-switching, as the current from plot (C) attains the compliance level, the memory state *x* from plot (B) [memristor voltage *v*_*m*_ from plot (A)] reaches almost instantaneously the zero of the state evolution function in Equation (9) (the voltage of the only possible operating point *Q*_*m,B*_ of the threshold switch) for *i*_*m*_ = *I*_*c*_, keeping unaltered throughout the compliance phase.

^32^Despite the input frequency is not close to 0 Hz in this case, as far as our micro-scale device is concerned, the input voltage waveform of Figure 18A may be practically considered a quasi-DC stimulus.

^33^No data point exists along the vertical line numbered 2 (5), which is simply meant to visualize the abrupt device set (reset) transition, and features the same slope as the 3 load lines drawn in Figure 20B to demonstrate, once more, that only their crossings with PDR parts of the device DC characteristic appear also as quasi-static points along the blue solid curve.

^34^As indicated for four *V*_*s*_ values in (a), the intersections between a load line and the device DC locus appear as quasi-DC points along the blue solid curve if and only if these crossings lie along either PDR branch or along the stabilized parts of the NDR branch of the device DC locus. Finally, numbered arrows and data-free set/reset transitions, featuring the same slope as the respective load lines, are shown only in plots (A) and (B), since, given the increasingly short horizontal distance between the blue square markers as *R*_*s*_ is assigned larger values, they would create clutter in plots (C) and (D).

^35^It is instructive to observe that even the ion channels in the human heart (Zhang et al., 2020) are volatile locally active memristors.

## Appendix: Microscale Device Structure and Fabrication Details

This section provides details on the fabrication and electro-forming process of our bi-layer structures. Figures 24A,B, respectively, show an illustrative sketch and a transmission electron microscope (TEM) image of the device stack, respectively. The substrate, consisting of a 100-nm-thick silicon dioxide (SiO_2_) layer, was grown over the silicon wafer by wet oxidation. A 4nm titanium (Ti) adhesion layer was then interposed between the substrate and a 20 nm-thick film of platinum (Pt), which was deposited by DC magnetron sputtering, forming a laterally unstructured bottom electrode. This electrode was found to feature a root mean square surface roughness level way lower than 1nm through atomic force microscope (AFM) measurement. A 21−nm-thick niobium oxide double layer, made up of a stoichiometric film placed over a sub-stoichiometric film, was then deposited on top of the bottom electrode. The oxygen content in the sub-stoichiometric layer was controlled by modulating the argon (Ar)-to- oxygen (O) mass flow ratio as reactive DC magnetron sputtering was carried out on a metallic niobium (Nb) target under a constant total pressure of 1.1 · 10^−3^mbar, and at room temperature. This low-temperature manufacturing step prevents the re-crystallization of the platinum medium, thus preserving a very smooth interface between the bottom electrode and the sub-stochiometric layer. The stoichiometric layer was also grown through the same manufacturing step. A circular top electrode, featuring a diameter between 20 and 100nm, was finally grown via electronic-beam evaporation of a 50nm-thick platinum layer through a shadow mask (Mähne et al., 2013). The virgin state device was subject to an electro-forming process to allow the first formation of a filament of high conductivity bridging the layer stack, which would otherwise naturally isolate the two electrodes. This forming step entailed the application of a purely negative voltage signal, ramping down step-wise in a quasi-static fashion from the null value, across the series combination between the memristor and a 330 Ω linear resistor. The role of the resistor is to limit the maximum current, which may ever flow through the micro-structure, preventing an irreversible physical damage to its layer stack. As the quasi-static voltage stimulus attained a value of about −6 V, the insulating layer of the virgin state device was found to experience a soft breakdown, which caused an abrupt upsurge in the memristor current, with a consequent increase in the voltage across the resistor. The current was found to increase up to a value of about 14 mA, while, due to the voltage divider effect, the memristor voltage descended and settled to a value as low as about 1.3 V, which precluded the hard breakdown of the bi-layer microstructure. Following the electro-forming step, the micro-device was found to undergo a reproducible cycle of consecutive abrupt off-to-on and on-to-off resistance switching processes upon the application of a *unipolar* quasi-static periodic signal of appropriate amplitude, well below the aforementioned forming value, between the series combination between the memristor and a suitable linear resistor. The increase and decrease in the conductivity of the filament within the niobium oxide bi-layer during the ascending and descending phase of the modulus of the quasi-static stimulus, respectively, originates from a Frenkel-Poole conduction mechanism, which, over the course of both the set and reset resistance switching transitions, is favored by thermal feedback effects. In particular, the progressive escalation in the temperature within the filament during the turn-on process poses serious risks for the lifetime of the micro-structure, calling for the adoption of strategies to limit the current upsurge over the course of the set transition. As mentioned in the paper, our studies on the device internal temperature during the turn-on phase ruled out a causal relationship between the insulator-to-metal Mott phase transition (IMT) mechanism and the threshold switching phenomenon (Slesazeck et al., 2015) at reasonably low currents (below 15 mA). It is in fact worth to mention that recent research studies have unveiled that, at comparatively higher currents, a device of this kind may undergo further threshold switching phenomena induced by the IMT mechanism (Kumar et al., 2017). Furthermore, under the low current regime, our niobium oxide threshold switching micro-device may be classified as a *volatile* memristor, since it is unable to store at least two stable states under zero input (Chua, 2015). Most importantly, as discussed in depth in this paper, the NaMLab micro-structure is blessed with the capability to enter the LA and EOC operating regimes, which makes it particularly attractive for the design of novel biomimetic neuromorphic circuits and brain-inspired computing systems.
